# Evolution of Metallo-β-lactamases: Trends Revealed by Natural Diversity and *in vitro* Evolution

**DOI:** 10.3390/antibiotics3030285

**Published:** 2014-07-01

**Authors:** María-Rocío Meini, Leticia I. Llarrull, Alejandro J. Vila

**Affiliations:** Institute of Molecular and Cellular Biology of Rosario (IBR, CONICET-UNR) and Biophysics Area, Faculty of Biochemistry and Pharmaceutical Sciences, National University of Rosario, Ocampo y Esmeralda, CONICET-Rosario, Rosario 2000, Argentina

**Keywords:** resistance, metallo-β-lactamases, evolution

## Abstract

The production of β-lactamase enzymes is one of the most distributed resistance mechanisms towards β-lactam antibiotics. Metallo-β-lactamases constitute a worrisome group of these kinds of enzymes, since they present a broad spectrum profile, being able to hydrolyze not only penicillins, but also the latest generation of cephalosporins and carbapenems, which constitute at present the last resource antibiotics. The VIM, IMP, and NDM enzymes comprise the main groups of clinically relevant metallo-β-lactamases. Here we present an update of the features of the natural variants that have emerged and of the ones that have been engineered in the laboratory, in an effort to find sequence and structural determinants of substrate preferences. This knowledge is of upmost importance in novel drug design efforts. We also discuss the advances in knowledge achieved by means of *in vitro* directed evolution experiments, and the potential of this approach to predict natural evolution of metallo-β-lactamases.

## 1. Introduction

The principal and more distributed resistance mechanism towards β-lactam antibiotics is the production of β-lactamases, enzymes capable of hydrolyzing the β-lactam ring characteristic of this class of antibiotics [1,2]. The hydrolyzed β-lactam antibiotic can no longer inhibit its target, the transpeptidase domain of Penicillin Binding Proteins (PBPs). Two general types of β-lactamases are known at present, serine-β-lactamases (SBLs) and metallo-β-lactamases (MBLs) [3].

The designation of one class of these enzymes as serine-β-lactamases stems from the fact that they possess an essential serine residue in their active site which is the nucleophile that attacks the β-lactam ring in the first step of the catalytic mechanism, resulting in the formation of a covalent acyl-enzyme adduct [1]. The serine-β-lactamases were historically characterized for being highly specialized towards penicillins or cephalosporins [1]. However, since the 1990s the number of variants known as extended spectrum β-lactamases (ESBLs) has significantly increased and ESBLs have disseminated among the Enterobacteriaceae species and *Pseudomonas* [4]. These enzymes include variants of the serine-β-lactamases TEM (named after the patient, Temoniera, from whom it was isolated), SHV (for sulphydral variable type 1), CTX-M (since it is active on cefotaxime, and first isolated in Munich) and OXA (oxacillinases) and are capable of hydrolyzing penicillins, first-, second-, and third-generations cephalosporins, and aztreonam. For these reasons it became a necessity to preserve carbapenems as the last resort antibiotics for the treatment of resilient infections. Nevertheless, in the last ten years genes coding for carbapenemases have rapidly spread in Enterobacteriaceae species and non-fermentative gram-negative bacteria [5]. The most remarkable carbapenemases are the serine-β-lactamases KPC (*Klebsiella pneumoniae* carbapenemase) and OXA, and the metallo-β-lactamases [5].

In contrast to SBLs, all metallo-β-lactamases are able to hydrolyze carbapenems and are not inhibited by the serine-β-lactamase inhibitors [6]. The latter is due to profound differences in the catalytic mechanisms displayed by these two classes of enzymes. At the base of the mechanistic differences is the fact that MBLs are Zn-dependent hydrolases, and present either one or two metal ions in their active site. A water molecule activated through coordination to one of the Zn(II) ions is thought to be the nucleophile involved in the first step of the mechanism of hydrolysis of β-lactams [7,8]. 

A group of metallo-β-lactamases are encoded chromosomally mainly in environmental bacteria or opportunistic pathogens, such as BcII from *Bacillus cereus* [9], GOB1 from *Elizabethkingia meningoseptica* [10], and L1 from *Stenotrophomonas maltophilia* [11]*.* However, the clinically relevant metallo-β-lactamases are encoded in mobile genetic elements and include VIMs (Verona Integron-encoded Metallo-β-lactamase) [12], IMPs (Imipenemase) [13], and the more recently emerged NDMs (New Delhi Metallo-β-lactamase) [14]. Though different inhibitors have been tested *in vitro*, there is no clinical drug able to inhibit any of the metallo-β-lactamases [15]. These metallo-β-lactamases show an extended substrate spectrum, including not only carbapenems, but also penicillins and the last-generation cephalosporins. Though they do not hydrolyze aztreonam [16], they are usually co-expressed with serine-β-lactamases that diminish the sensibility of the bacteria towards this compound [1].

The mobile genetic elements that harbour genes for carbapenemases usually also contain genes that confer resistance towards other classes of antibiotics, such as aminoglycosides and quinolones, giving rise to multi-drug resistant strains [14]. The only remaining therapeutic options to treat infections caused by these strains are the antibiotics tigecycline and colistin, which are not always useful and, on top of that, not innocuous (for example, colistin is nephrotoxic) [17,18]. This situation has led many authors to anticipate a post-antibiotic era, a situation that would push all the medical system into crisis [18,19]. The inadequate use of antibiotics favors selection of resistant bacteria, and the rapid intra- and inter-species dissemination of resistance mechanisms renders each new antibiotic that is commercially introduced ineffective in very short terms [1,2,20]. In light of the fast adaptation displayed by bacteria to antibiotic pressure, it would be a great advantage to count with tools that would allow us to anticipate the evolution of β-lactamases under different clinical situations. In the case of TEM serine-β-lactamases, 217 variants (Lahey database [21], February 2014) have been found in the clinics and distinct phenotype groups are distinguished according to the substrate spectrum and inhibitor sensibility [22]. Variants with highly incremented activity towards different cephalosporins or displaying inhibitor resistance were found only a couple of years after the FDA (USA Food and Drug Administration) approval of these compounds [23]. In parallel, different directed molecular evolution experiments have allowed the identification of improved variants with mutations that were later found naturally, proving the potency of these experiments to predict future variants of SBLs in response to the use of new antibiotics or inhibitors [23,24,25,26]. Directed molecular evolution experiments have been much less exploited to predict MBL variants with improved catalytic efficiency. The metallo-β-lactamase BcII, which is not produced by a clinically relevant pathogen, could be a precursor of the more efficient MBLs present in bacteria that are exposed to increasing amounts of antibiotics [27,28]. Under this hypothesis, BcII would be a unique template to explore *in vitro* evolution of enhanced levels of resistance through the potential improvement of its catalytic efficiency and substrate spectrum. A directed evolution experiment performed with BcII gave rise to evolved variants towards a previously poor substrate [28,29]. This directed evolution assay identified a mutation that has been selected naturally among the IMP enzymes, which indeed confers a wider substrate profile [27,30]. The rationalization of the results from directed evolution experiments in MBLs is not trivial, since evolved variants are selected based on their ability to allow bacteria to survive in the presence of increasing concentrations of antibiotics (an increased Minimal Inhibitory Concentration, MIC,value) whilst our attempts to comprehend how mutations affect the ability to confer resistance generally involve assays with purified recombinant proteins. The conditions that we screen to achieve the overexpression of fully active recombinant enzymes most likely do not mimic the more physiological conditions under which MIC values are determined. For MBLs in particular, which fold in the periplasm after being translocated through the cytoplasmic membrane [31], metal availability at the time of refolding can be critical for achievement of full-blown activity *in vivo* [32,33]. 

In the following sections, we will describe the known emerged variants of the most important MBL groups, VIMs, IMPs, and NDMs. Also we will include variants obtained *in vitro* by methods like codon randomization and we will summarize the lessons learned from directed molecular evolution experiments. Some of these topics were already discussed [34,35,36], but we will focus our attention on the link between mutations and substrate spectrum changes.

Although reports of the epidemiology of MBLs are increasing [37,38], there is still an important knowledge gap regarding the important reaction mechanisms and structure-function properties of MBLs, and more in-depth studies are required to address this and to acquire mechanistic insights that will ultimately support novel drug design efforts. In this review we focus mainly in the sequence and structural determinants of function and of substrate preferences: residues that modulate the activity profile and the mobile loops flanking of the active sites of MBLs that are important in recognizing a broad repertoire of substrates. A comparative analysis of the MBL variants is, in most cases, complicated by the differences found in the assay conditions, as will be highlighted throughout the review. The establishment of consensus protocols for MIC and enzymatic studies is imperative for the scientific community to succeed in understanding the mechanisms underlying the differences between the MBL variants.

Standardized nomenclature and reference sequences were taken from the Jacoby and Bush website database (Lahey database [21]). The standardized class B β-lactamase (BBL) numbering scheme proposed by Galleni, Dideberg, and others is employed for numbering residue positions [39,40]. An automated database, Metallo-β-lactamase Engineering Database (MBLED), built by Oelschlaeger and co-workers, was also taken as reference for MBL sequences [36].

## 2. Metallo-β-lactamases: Promiscuous Traits and Conserved Active Sites Endowing Rapid Adaptation

**Figure 1:**
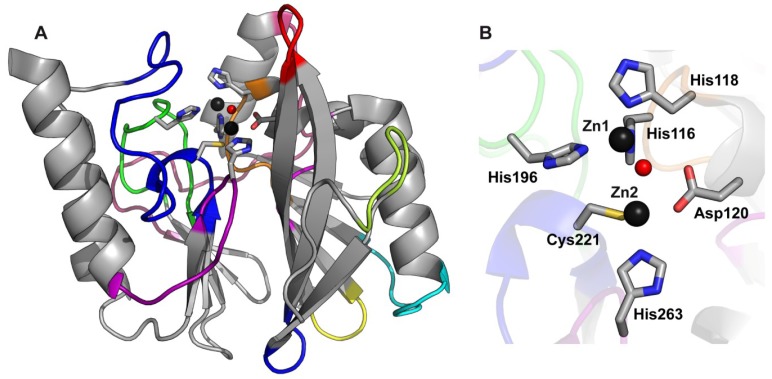
B1 MBLs conserved global fold and active site. (**A**) The crystallographic structure of VIM-2 (PDB 1KO3) is shown as an example of the conserved MBLs fold; (**B**) The active site of VIM-2 is shown as an example of B1 enzymes, consisting of two Zn(II) ions (dark gray spheres) bound by the indicated ligand residues (shown as sticks) and a bridging water/hydroxide molecule (red sphere).

Metallo-β-lactamases display a very low sequence identity, as low as 10% in some cases, and are hence divergent proteins, which, nonetheless, share a general fold and a zinc binding motif in the active site [3,40]. Most of the conserved sequence features correspond to residues that are Zn(II) ligands. The common scaffold is a αβ/βα sandwich (Figure 1), with the active site situated within a shallow groove formed at the interface of the two αβ domains [6]. Given the structural similarity between the two halves of the protein, it has been proposed that MBLs may have arisen from a gene-duplication event. However, no possible candidate ancestors meeting this requirement have been identified yet. Instead, there is solid evidence that metallo-β-lactamases belong to an ancient superfamily of metallo-hydrolases with diverse functions, known as the MBL superfamily [41]. The αβ/βα fold and a metal binding motif in the active site are highly conserved across the MBL superfamily, which is considered to have evolved billions of years ago [41]. Despite the fact that members of this superfamily are able to bind two metal ions (Zn(II), Fe(II), Mn(II), Mg(II)), the metal ligands present in MBLs are unique to enzymes with lactamase activity, revealing that this metal binding motif has been selected for beta-lactam hydrolysis [32]. Indeed, these ligands rarely tolerate changes without compromising activity [42,43,44]. 

Three MBL subclasses are defined based on sequence and structural alignments: B1, B2, and B3 [39,40]. The clinically relevant metallo-β-lactamases belong to subclass B1 and share approximately 23% amino acid sequence identity. B1 and B3 metallo-β-lactamases, on one hand, are broad spectrum enzymes, being able to hydrolyze penicillins, cephalosporins, and carbapenems with a wide variety of hydrolytic efficiencies *in vitro* and with a broad range of resistance profiles *in vivo* [3]. They require two Zn(II) ions bound to their active sites. B1 enzymes display a conserved metal binding motif with ligands located in protein loops (H_116_-X-H_118_-X-D_120_ from Loop-L7, H_196_ from Loop-L9, C_221_ from Loop-L10 and H_263_ from Loop-L12). As depicted in Figure 1, for the B1 enzyme VIM-2, the coordination sphere of the metal in the Zn1 site involves three His residues (H_116_, H_118_, and H_196_) and a water/hydroxide molecule arranged in a tetrahedral geometry, while the metal in the Zn2 site adopts a trigonal bipyramidal coordination sphere, with D_120_, C_221_, H_263_, a water molecule, and the formerly mentioned water/hydroxide molecule as ligands [45,46,47]. The water/hydroxide molecule is a bridging ligand between the two metal sites and it would detach from the metal ion at the Zn2 site prior to nucleophilic attack on the carbonyl carbon of the β-lactam ring [7,8]. The Zn1 site of B3 enzymes closely resembles that of B1 enzymes, however, the B1 Zn2 ligand Cys221 is replaced by His121 in B3 enzymes, and a third water molecule participates as a fifth ligand giving rise to a square pyramidal coordination sphere of Zn2 [48]. B2 metallo-β-lactamases, on the other hand, are exclusive carbapenemases and are active with one Zn(II) ion bound to the Zn2 site (superimposable to the B1 Zn2 sites), whilst the second Zn(II) ion has an inhibitory effect [49]. 

Here we will cover the evolutionary features of the most clinically relevant enzymes (the VIM, IMP and NDM families), all belonging to subclass B1. Thus, the metal binding sites of all cases here discussed are identical to that depicted in Figure 1. Given that the same scaffold and the same metal binding site can serve for a diversity of substrate specificities, mutations shaping the substrate profile can be located either: (1) in the loops flanking the active site, which define the enzyme cavity; or (2) in second sphere residues. 

The role of protein dynamics is essential in shaping MBL function, given their broad substrate spectrum. Several studies of molecular dynamics on resting-state and substrate-bound MBLs are available [29,50,51,52,53]. These simulations pointed out the role of the dynamics of Loops L3 and L10 upon substrate binding in the picosecond-nanosecond time scale [29,51,52]. NMR experiments, instead, have been less exploited in MBLs. NMR has shown that Loop-L3 has greater flexibility on the picosecond-nanosecond time scale than the whole molecule [54]. ^19^F-NMR spectroscopy was employed with NDM-1 to follow the conformational changes in Loop-L3 upon binding of different inhibitors [55]. However, NMR can provide information on dynamics at different time scales, including the microsecond-millisecond time scale, which is in the range of catalysis and binding events. This still represents an unexplored and promising field to be exploited yet. 

**Figure 2:**
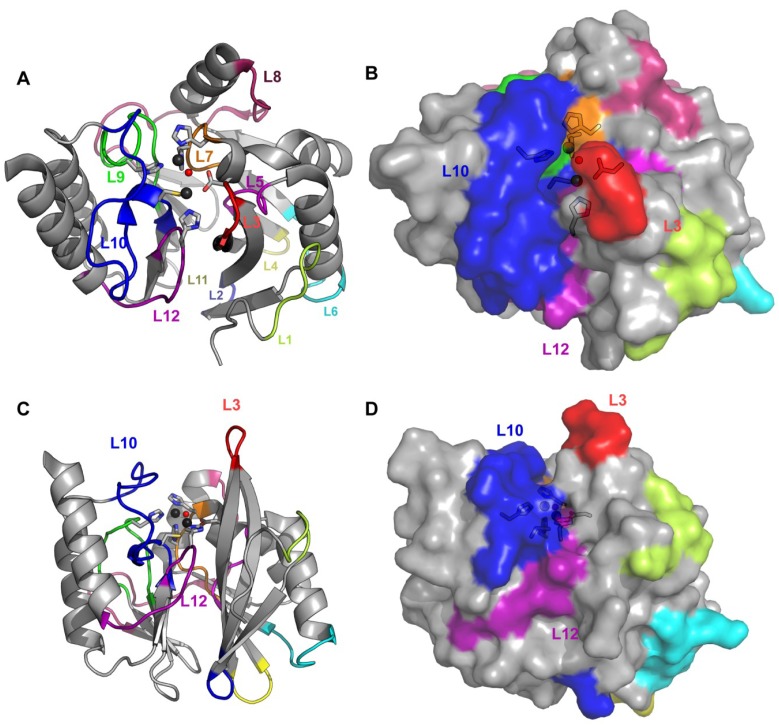
Loops and active site of B1 enzymes. (**A**) The loops, conserved in most of B1 enzymes, are marked in the crystallographic structure of VIM-2 (PDB 1KO3) in cartoon representation; (**B**) The same angle is shown as surface to distinguish the active site cavity; (**C**) and (**D**) Cartoon and surface representations viewed from another angle to show the active site walls formed by Loops L10 and L3 and the floor constituted by Loop-L12.

Second sphere residues involve a network of hydrogen bonds below the active site base, which indirectly affect the electronic structure and geometry of the Zn(II) ions [56]. Figure 2 depicts the loops defining the active site. These loops contain: (1) Zn(II) ligand residues, arising from Loops L7, L9, L10 and L12; (2) second-sphere ligands, from Loops L3, L5, L10, and L12; and (3) residues which interact with substrate moieties through hydrophobic contacts, located in the two protruding Loops, L3 and L10.

## 3. VIM Variants

**Figure 3:**
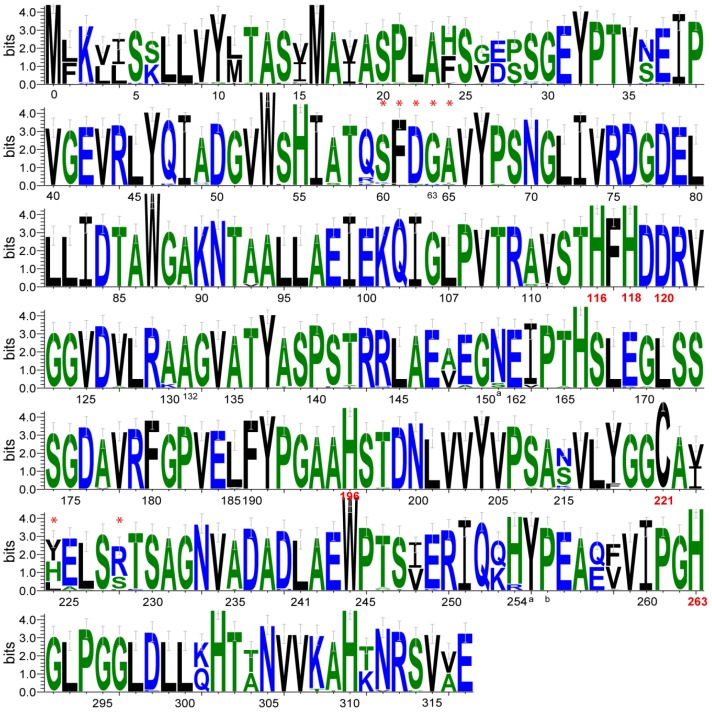
Logo representation of the alignment of the VIM variants. Logo was performed with WebLogo3 [69]. The compositional adjustment used was the typical amino acid usage pattern for proteins. Sequence is numbered according to the BBL scheme. The position of the amino acids that are Zn(II) ligands is highlighted in red. Positions discussed in the text are marked with an upper asterisk.

**Figure 4:**
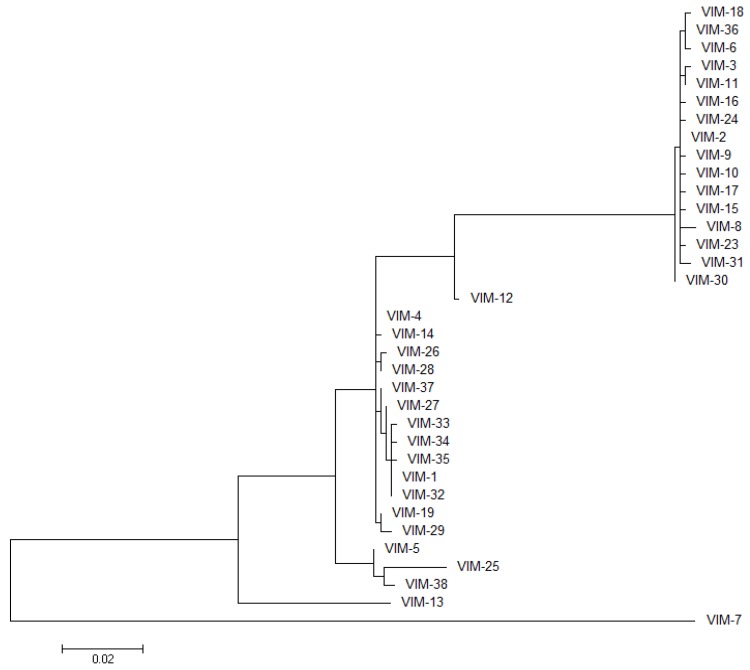
Molecular phylogenetic analysis of VIM variants. The evolutionary history was inferred by using the Maximum Likelihood method based on the Tamura-Nei model [70]. The tree with the highest log likelihood (−2610.7954) is drawn to scale, with branch lengths measured in the number of substitutions per site. The analysis involved 35 nucleotide sequences. Evolutionary analyses were conducted in MEGA6 [71].

VIM-1 was identified in 1999, in an Italian clinical isolate of *P. aeuruginosa* resistant to carbapenems [12]. Soon after, the variant VIM-2 was reported, arising from an earlier clinical isolate from 1996 in France, [57]. Since then, 40 different VIM variants emerged worldwide (up to February 2014) [21]. Among them, VIM-2 is by far the most prevalent clinical variant as well as the one with the largest geographical spread [58]. VIM variants present sequence similarities ranging between 81% and 99.6% (72.9%–99.6% sequence identity, including VIM-7, the most divergent variant; 87.7%–99.6% identity without VIM-7; Figure 3 and Supplementary File 1), and show a directional and monophyletic mode of evolution with formation of two major clusters: VIM-1 and VIM-2 [35]. An updated phylogenetic tree of the VIM family is shown in Figure 4. The most divergent variant for the VIM-1 cluster is VIM-7. Despite the large amount of VIM alleles and their clinical relevance, at the moment, only 8 VIM variants have been characterized both biochemically and in terms of their resistance profiles: VIM-1 [12,59], VIM-2 [57,59], VIM-4 [60], VIM-7 [61], VIM-11 [62], VIM-13 [63], VIM-19 [64], and VIM-31 [65]. Crystal structures are available for VIM-2 in the resting state [46] and in complex with an inhibitor [66], VIM-4 [60] and VIM-7 [67]. The most relevant mutations found along the VIM family are located in the two loops flanking the active site: L10 and L3.

**Figure 5:**
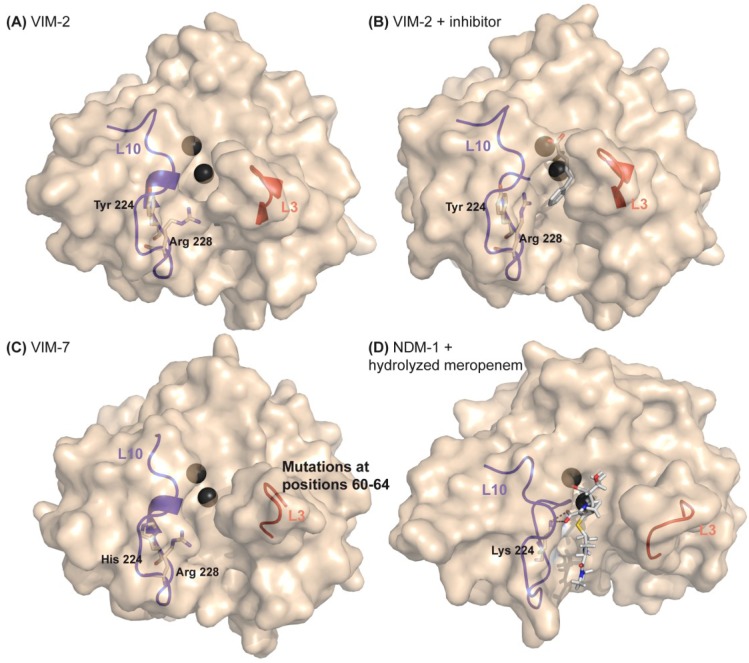
Structural features related to substrate preferences on the VIM variants family. (**A**) Crystallographic structure of VIM-2 (PDB 1K03); (**B**) Crystallographic structure of VIM-2 bound to a mercaptocarboxylate inhibitor (PDB 2YZ3); (**C**) Crystallographic structure of VIM-7 (PDB 2Y87); (**D**) Crystallographic structure of NDM-1 bound to hydrolyzed meropenem. The structures are represented as surface, Zn(II) ions are shown as dark grey spheres, Loop-L10 and Loop-L3 are shown as cartoon representations, residues at positions 224 and 228 are shown as sticks in VIMs structures. Lys224 is shown as stick in the NDM-1 structure, interacting with the C3-carboxylate of hydrolyzed meropenem. Arg228 has been proposed to act as a Lys224 equivalent on VIMs. Arg228 present different conformations in different VIM structures and is capable of adjusting its position to accommodate the inhibitor’s phenyl group, being stabilized by the interaction between the carbonyl group of Ala231 with the hydroxyl group of Tyr224. The binding cleft of VIM-2 is slightly narrower than that of VIM-7 due to Phe61 (Leu in VIM-7) and the different conformation of Arg228.

Loop-L10 is a long loop defining one of the active site walls (Figure 2 and Figure 5) that span from residues 221 to 241, where most of the key mutations in the VIM family are found. In other B1 enzymes, a conserved Lys residue is found at position 224, which is known to guide substrate binding by interaction with the conserved carboxylate moiety of the antibiotics, together with the interaction with the Zn2 site [6,47,68]. VIM lactamases display His (39%, including VIM-1, VIM-4, and VIM-7), Tyr (44%, as in VIM-2 and related) or Leu (17%, including VIM-13) residues at position 224 (Figure 3). Instead, an Arg residue is mostly found in the nearby position 228 in 70% of the VIM alleles, which is supposed to play the role of carboxylate steering in this family of enzymes [46,66,67].

**Figure 6:**
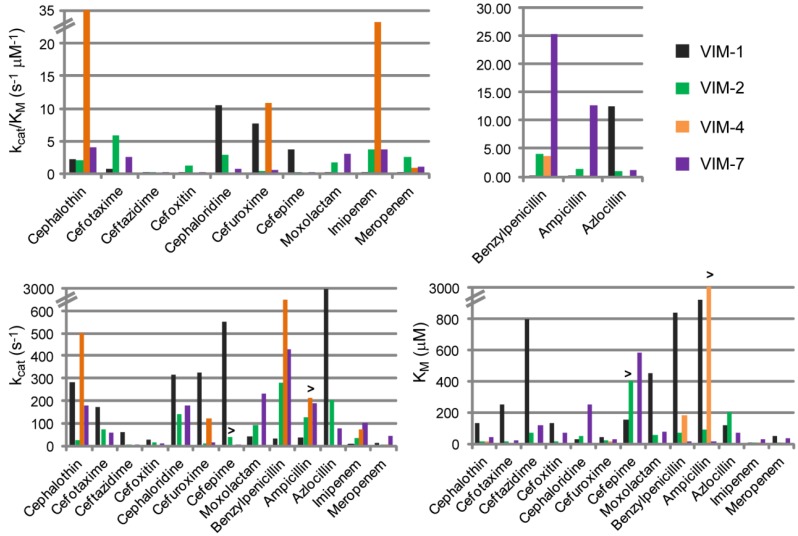
Kinetic parameters for VIM-1, VIM-2, VIM-4 and VIM-7 variants towards various β-lactam antibiotics. The data for VIM-1 and VIM-2 correspond to Docquier *et al.* [59] and the conditions employed for the reactions were 50 mM Hepes pH 7.5, 50 μM ZnCl_2_, and 20 μg/mL BSA, 30 °C. The data for VIM-4 correspond to Lassaux *et al.* [60] and the conditions employed for the reactions were 15 mM Hepes pH 7.2, 50 µM ZnCl_2_ and 20 µg/mL BSA. The data for VIM-7 correspond to Samuelsen *et al.* [61] and the conditions employed for the reactions were 50 mM sodium cacodylate pH 7, 100 μM ZnCl_2_, and 0.1 mg/mL BSA, 25 °C.

In general, all VIM enzymes show a broad substrate spectrum toward penicillins, cephalosporins, and carbapenems. VIM-1 and VIM-2 differ by 17 residues, three of them at positions 223, 224, and 228 located at the vicinity of the active site in Loop-L10 (Figure 2 and Figure 5). The group of Rossolini and coworkers performed a comparative kinetic study of VIM-1 and VIM-2 at exactly the same reaction conditions [59]. VIM-2 displays better hydrolytic efficiencies towards penicillins than VIM-1, an effect that is particularly enhanced for benzylpenicillin and ampicillin (Figure 6). Instead, VIM-1 is only clearly superior to VIM-2 against azlocillin. In the case of cephalosporins, VIM-2 performs better than VIM-1 towards cefalothin, cefoxitin, cefotaxime, and moxalactam, while VIM-1 performs better than VIM-2 towards cefaloridine, cefuroxime and cefepime. Both VIM-1 and VIM-2 are efficient carbapenemases, with low *K*_M_ and *k*_cat_ values, a fact that distinguishes VIM enzymes from other MBLs, which achieve comparable hydrolytic efficiencies against carbapenems as a combination of high *K*_M_ and high *k*_cat_ values [59]. These differences in enzymatic activities are only directly correlated to differences on MIC values for the case of cefepime [12,57]. However, it should be noted that different expression vectors and *E. coli* strains were used for the MIC assays, and therefore minor differences might be masked due to other factors, such as protein expression levels. It is likely that these differences are mostly due to changes at positions 224 and 228 (His/Ser in VIM-1 and Tyr/Arg in VIM-2) (Figure 5). VIM enzymes lack the conserved Lys224 in B1 enzymes, which interacts with the carboxylate moiety on C4 or C3 of the substrate [72,73]. Then, Arg228 in VIM-2 has been proposed to replace Lys224 on this interaction [46] (Figure 5). The VIM-2 structure reveals that the hydroxyl group of Tyr-224 interacts with the amide nitrogen of Gly 232 and, through a water molecule, with the carbonyl oxygen of Asn233 and with the Nδ1 of the Zn1 ligand His196 [46].

No structure is available yet for VIM-1. However, the structure of VIM-4, which differs from VIM-1 only by mutation S228R, is very instructive [60]. VIM-4 displays better catalytic efficiencies for benzylpenicillin than VIM-1 (as VIM-2 does), but worse for ampicillin (Figure 6). With cephalosporins, *K*_M_ values are lower than those for VIM-1 in general and similar to VIM-2 values. In particular, for cephalotin, the catalytic efficiency was higher than VIM-1 and VIM-2. Regarding carbapenems, imipenem, and meropenem are better hydrolyzed by VIM-4 with respect to VIM-1. Notably, VIM-4 was even better than VIM-2 against imipenem [60]. Overall, the hydrolytic profile of VIM-4 is closer to VIM-2 than to VIM-1, even though in sequence it differs by 16 and 1 residues, respectively. This can be attributed to the fact that both VIM-2 and VIM-4 possess an Arg residue at position 228, while Ser228 in VIM-1 would give rise to a more relaxed cavity (Figure 5). This hypothesis is supported by the fact that VIM-19 (with His224/Arg228, like VIM-4) shows a similar substrate profile, with a somehow increased efficiency towards carbapenems [64]. 

VIM-13 displays impaired catalytic efficiencies and resistance profiles against ceftazidime and cefepime with respect to VIM-1 [63]. VIM-13 differs from VIM-1 by 19 mutations distributed all along the primary sequence. Two of them correspond to residues located in Loop-L10: H224L and S228R. Bou and coworkers proved that single and double mutants at these positions in VIM-13 play a role on substrate preferences [63]. VIM-1 yielded ceftazidime and cefepime MICs of >256 and 64 mg/L for *E. coli*, respectively, and VIM-13 yielded MICs of 6 and 4 mg/L, respectively. Leu224His-VIM-13 showed minor variations on MIC values. In contrast, a substantial increase on the MIC against ceftazidime was observed by single mutation R228S in VIM-13. Interestingly, the double mutant L224H/R228S-VIM-13 displayed MIC and *k*_cat_*/K*_M_ values against ceftazidime and cefepime which compare to those of VIM-1. This study leads to two important conclusions: (1) the effects of these two positions are related, as stems from the synergistic effect observed on the MIC values; and (2) these two changes are enough to mimic the cephalosporin substrate profile of VIM-1, despite the existence of differences in other 17 residues differing between these two enzymes.

Therefore, Arg228, when present in VIM variants, seems to be largely responsible for the substrate profile: the narrower cavity of variants displaying Arg228 would explain their higher affinity and better catalytic efficiencies towards some substrates and at the same time explain the exclusion of substrates with bulky, containing a positively charged cyclic substituent (R2), like cefepime and ceftazidime.

Loop-L3 is a short loop present in most B1 MBLs, encompassing residues 60–66 (Figure 2). This loop is usually flexible, and can close upon ligand binding [74], generally providing hydrophobic contacts with the substrate through one aromatic residue [47,75]. The flexibility of this loop has been probed by NMR spectroscopy in several B1 enzymes [76,77], and its position is undefined in most crystal structures due to the absence of well-ordered electron density in this region, which depends mostly on the crystal symmetry [78]. Almost all VIM variants present a fully conserved sequence at Loop-L3, with a Phe residue at position 61. VIM-7, the most divergent variant, has been studied by Samuelsen, Spencer and co-workers [61,67]. This enzyme presents various substitutions at this loop, including S60K, F61L S62G, G63D, and A64T (mutations are compared to the VIM-2 sequence) (Figure 3 and Figure 5c; Supplementary File 1). Also, at the base of the Loop-L3 in position 68, a conserved Pro residue in VIM variants is replaced by a Ser residue in VIM-7. In Loop-L10, VIM-7 possesses His224/Arg228 (resembling VIM-4). VIM-7 displays higher catalytic efficiency for penicillins than VIM-1 and VIM-2. As with the other variants containing Arg228, it displays lower efficiency against cephalosporins containing a positively charged cyclic substituent in respect to VIM-1, being even worse than VIM-2 [67]. The VIM-7 structure shows that Loop-L3 is positioned farther from the active site with respect to VIM-2 and VIM-4, resulting in a more open active site (Figure 5c). Substitutions on this loop may collectively alter the dynamics of this region and influence substrate specificity. The replacement of Pro68Ser at this position may increase the flexibility of the loop contributing to the altered activity profile. The presence of His224 in VIM-7 disrupts the hydrogen-bond network in the active site that implicates Tyr224 on VIM-2. In VIM-7, His224 does not make equivalent interactions and gives rise to a slightly more positively charged binding pocket for VIM-7 compared with VIM-2, which might further worsen the binding of positively charged cephalosporins. 

A saturation mutagenesis approach was performed on VIM-2, by Docquier and coworkers, in order to analyze the influence on activity of some of the residues at Loop-L3 [79]. One of these residues was Ala64, which represents a structural equivalent of the mobile flap Trp64 in the IMP lactamases (see below). The other positions were Phe61 and Tyr-67 from Loop-L3 and Trp87 from Loop-L5, which constitute a hydrophobic patch hypothesized to play a role on substrate binding. Moreover, the importance of residues 61 and 67 for ligand binding was demonstrated from the structure of VIM-2 in complex with a mercaptocarboxylate inhibitor [66]. Nevertheless, the positions at Loop-L3 tolerated several amino acid substitutions without a significant impact on the MIC values. In contrast, residue Trp87 in Loop-L5, which is conserved in the VIM variants, did not tolerate changes as observed by the drastic decreases on the MIC values of various substrates. The replacement of this residue by Phe or Ala did not affect catalytic efficiency; instead it affected stability and *in vivo* folding. 

In summary, during evolution, the substrate profile of VIM enzymes is largely determined by the synergistic effect of mutations at positions 224 and 228 (Table 1). The interplay between these two sites resembles that found in TEM lactamases, where a positively charged Arg is required for interacting with the substrate carboxylate, but can be found in different alternative positions [80]. How mutations at positions 224 and 228 of VIM variants modulate the substrate profile clearly deserves further attention. The situation of VIM-7, being more divergent, is still unclear, given that mutagenesis of Loop-L3 in VIM-2 has shown a large degree of tolerance to mutations. Instead, the fact that a conserved Pro residue located at the base of Loop-L3 at position 68 is replaced by a Ser in VIM-7, could be responsible for the substrate profile changes. Finally, further biochemical characterization of VIM enzymes in terms of their resistance profile is needed to assess the role of all these residues. However, it is mandatory to establish consensus protocols for MICs and enzymatic studies that allow a direct comparison of data reported by different laboratories. 

**Table 1:** Substrate profile of the different VIM (Verona Integron-encoded Metallo-β-lactamase) variants and mutations, with respect to VIM-1, that were proposed as responsible for the altered substrate profiles.

Variant	Substrate Profile, Compared to VIM-1	Total Number of Mutations, Compared to VIM-1	Mutations Compared to VIM-1, Responsible for Changes in Activities
**VIM-2**	Higher catalytic efficiency for penicillins Lower catalytic efficiency for cefepime	17	H224Y, S228R
**VIM-4**	Higher catalytic efficiency for benzylpenicillin but worse for ampicillin Higher catalytic efficiency for imipenem (and also higher than VIM-2)	1	S228R
**VIM-13**	Lower catalytic efficiency for ceftazidime and cefepime	19	H224L, S228R
**VIM-7**	Higher catalytic efficiency for penicillins (and also higher than VIM-2) Lower catalytic efficiency for ceftazidime and cefepime	61	S228R Mutations at Loop-L3: S60K, F61L S62G, G63D, and A64T

## 4. IMP Variants

**Figure 7:**
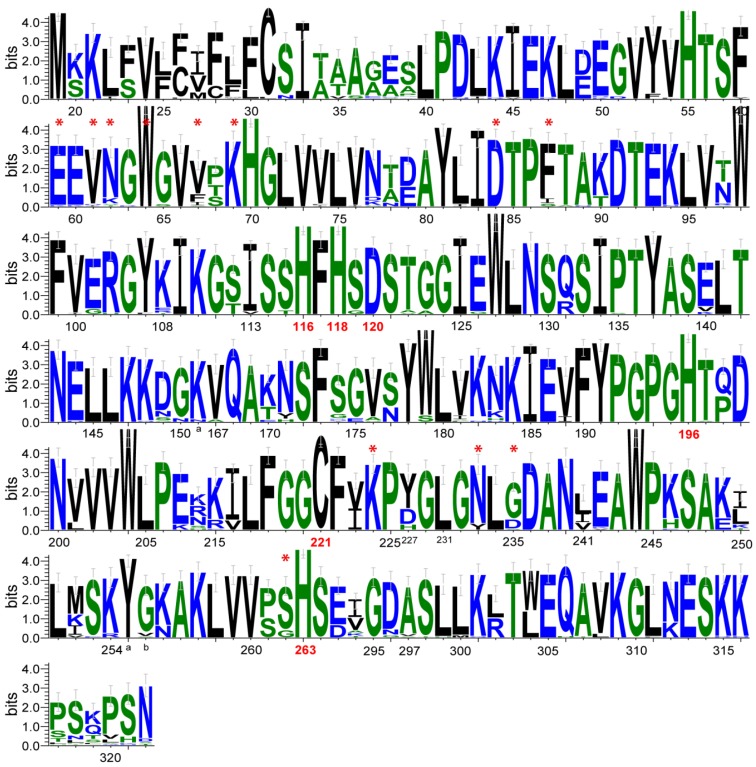
Logo representation of the alignment of the IMP variants. Logo was performed with WebLogo3 [69]. The compositional adjustment used was the typical amino acid usage pattern for proteins. Sequence is numbered according to the BBL scheme. The position of the amino acids that are Zn(II) ligands is highlighted in red. Positions discussed in the text are marked with an upper asterisk.

**Figure 8:**
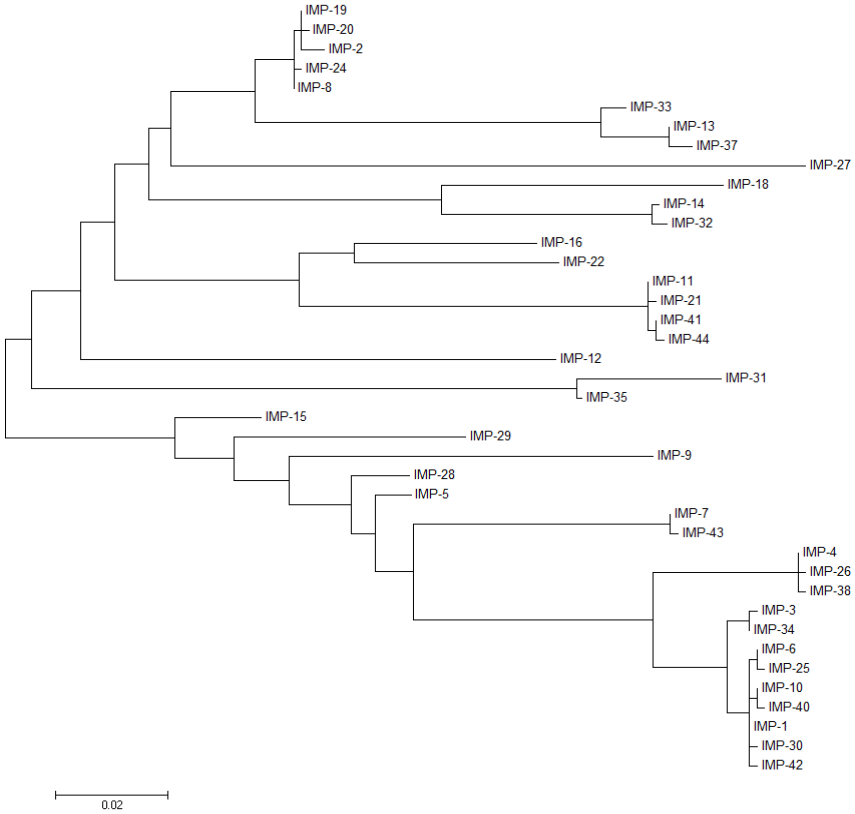
Molecular phylogenetic analysis of IMP variants. The evolutionary history was inferred by using the Maximum Likelihood method based on the Tamura-Nei model [70]. The tree with the highest log is drawn to scale, with branch lengths measured in the number of substitutions per site. The analysis involved 40 nucleotide sequences. There were a total of 738 positions in the final dataset. Evolutionary analyses were conducted in MEGA6 [71].

IMP was identified as the first transferable metallo-β-lactamase, coming from a clinical isolate of *Pseudomonas aeruginosa* collected in Japan in 1988 [81]. Alleles from the IMP family were then found in *Enterobacteriaceae* and distributed along Asia and the rest of the world [82]. Overall, the IMP variants present 85%–99.6% of sequence similarity (80%–99.6% sequence identity; Figure 7 and Supplementary File 2), giving rise to a diffused dendogram that suggests a polyphyletic origin of IMPs [35], contrasting with the VIM group. An updated phylogenetic tree of the IMP family is shown in Figure 8.

**Figure 9:**
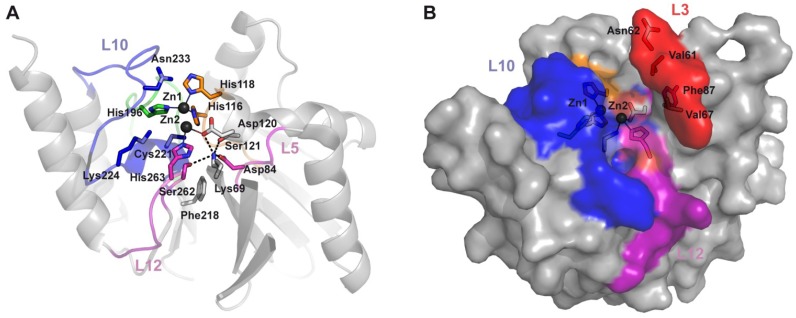
Structural features related to substrate preferences on the IMP variants family. (**A**) The active site residues are shown together with residues that accept variations but with substrate dependent effects on the randomization experiments performed by Palzkill *et al.* [44,73]. Since most of them are second shell residues, relevant hydrogen bond interactions are shown as dashed lines. Lys224 and Ser262 accept minimal variations. Zn(II) ions are represented as dark gray spheres; residues are showed as sticks; (**B**) Surface representation of IMP-1 structure (PDB 1DDK), showing the residues at Loop-L3 that accepted variations preserving hydrophobicity with some substrate dependence.

**Figure 10:**
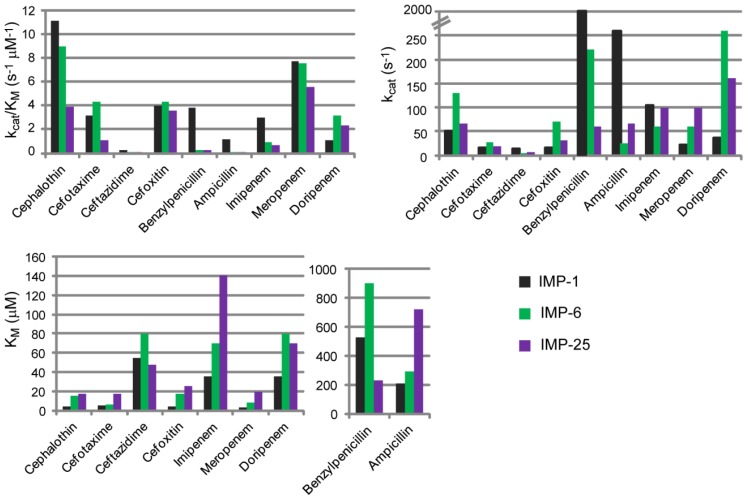
Kinetic parameters for IMP-1, IMP-6 and IMP-25 variants towards various β-lactam antibiotics. The data correspond to Liu *et al.* [83], the conditions employed for the reactions were Buffer MOPS pH 7, 100 µM ZnSO_4_, 10 µg/mL BSA, 30 °C.

Two major groups can be distinguished among IMP variants, based on the identity of the second sphere residue at position 262 (Figure 7 and Figure 9), which has been considered as responsible for tuning the substrate preferences [27]. IMP-1-like variants possess a Ser residue at position 262, while IMP-6-like variants present a Gly residue at the same position (IMP-6, IMP-3, IMP-12, IMP-25, IMP-27, and IMP-38). IMP-6 displays similar catalytic efficiencies to those shown by IMP-1 towards cefalotin, cefotaxime [13,27], cefoxitin, meropenem, and doripenem (Figure 10) [83]. However, IMP-1 is more efficient than IMP-6 towards penicillins (in particular penicillin G and ampicillin), ceftazidime, cephaloridine, and imipenem [84]. These differential activities are also reflected on the respective MICs on *E. coli* cells [27,30,83]. The first characterization of IMP-6 [30] against meropenem showed a marked increase in the MIC and catalytic efficiency compared to imipenem. Thus, it was hypothesized that mutation S262G would be an adaptation towards newer carbapenems, in detriment of penicillinase activity [30]. A more recent work on IMP-25 reported an increase in meropenem MIC for IMP-6 and IMP-25 [83], despite not as high as previously reported [30]. In fact, the catalytic efficiencies did not show the dramatic increment observed by Yano *et al.* [30]. These differences in the catalytic efficiencies can be due to the different conditions employed in the enzymatic assays (Yano *et al.*, 50 mM Phosphate Buffer pH 7.0 with 10 µM ZnCl_2_, and Liu *et al*., MOPS pH 7, 100 µM ZnSO4, 10 µg/mL BSA). Despite these differences, the *k*_cat_ values correlated with the meropenem MIC values, as both parameters gradually increased in the same direction: IMP-1→IMP-6→IMP-25. The *K*_M_ values were very low in all cases, suggesting that MBLs would be saturated at the MIC conditions [83].

Two possible scenarios have been proposed. On one side, IMP-6 might be an ancestor of IMP-1, and in this context, the role of mutation G262S would have been to broaden the substrate profile. On the other side, if IMP-1 is an ancestor of IMP-6, the effect of mutation S262G would have been to improve the activity towards newer carbapenems at the expense of the penicillinase activity. These hypotheses heavily rely on assumptions of the evolutionary timeline of these enzymes that cannot be verified. However, it is evident that position 262 on IMP enzymes effectively affects their substrate preferences.

At present, only structures for IMP-1 have been deposited, and the analysis of the impact of these mutations relies on molecular dynamic simulations [85]. Oelschlaeger and coworkers have proposed the existence of a “domino effect” involving the Zn(II) ligand His263, the residue at position 262, and one of the substituents in the substrates. Dissecting the different structural factors that involve second sphere residues and that modulate substrate preference is much more difficult than for the case of loops in the active site, since they do not involve direct interactions with the substrate and the impact is indirect. Despite further work is required to clarify some details, mutagenesis analysis at position 262 in the IMP enzymes clearly reveals that the hydrogen bond network below the active site indeed plays a crucial role in determining substrate specificities (a similar conclusion stems from studies on the B1 enzyme BcII, as described below) [84].

Codon randomization experiments on IMP-1 were performed by Palzkill and coworkers to evaluate the role of several residues near the active site cleft (Figure 9a) [44,73]. The selection criterion was based on the ability to confer resistance towards ampicillin [44]. A subsequent work extended the study to cefotaxime, imipenem and cephaloridine, showing that the requirements for some residues are highly dependent on the substrate [73]. The Ser residue at position 262 was shown to be essential for ampicillin, imipenem or cephaloridine, while a Gly substitution was found upon selection with cefotaxime. These results are in full agreement with the previously discussed studies on different natural IMP variants. As expected, mutations on the Zn(II) ligands are not tolerated. 

Regarding residues located in the loops that might participate in interactions with the substrates, in contrast to VIM enzymes, all IMP variants possess a Lys residue at position 224 in Loop-L10 (Figure 7 and Figure 9a), which interacts with the substrate carboxylate [47]. No changes in this residue were tolerated for ampicillin, imipenem and cefotaxime selection, but variant with the substitution K224Q was viable against cephaloridine. Mutant K224Q proved to be efficient in hydrolyzing cephaloridine and cefotaxime, but showed a reduced activity against ampicillin and imipenem. Mutations to Ala, Glu, and Arg at position 224 showed that *K*_M_ increases in general with neutral or negatively charged residues, indicating that a positive charge is required for an electrostatic interaction with the carboxyl moiety of the substrate (C-3 of penicillins or C-4 of cephalosporins) [86]. Asn233 is another residue fully conserved in Loop-L10 (Figure 9a), which is supposed to be involved in substrate binding and catalysis [47,87]. Despite this fact, this position was more tolerant to randomization than position 224, and several mutations were selected with different substrates [44]. The mutant N233A presents higher *k*_cat_/*K*_M_ values, not only for ampicillin, but also for other substrate tested, such as cephalosporins. Different amino acids were found at position 233 among mutants selected for cefotaxime, but the wild type residue was critical for imipenem resistance. For cephaloridine hydrolysis, a Glu residue was preferred at position 233. The IMP natural variants, IMP-14, -31, -32 and -35 show a substitution for a Tyr residue. A former study, in which N233 in IMP-1 was mutated to Ala and Asp, showed that the effects in kinetic parameters are substrate dependent. Cephalotin and benzylpenicillin were not affected, but the *k*_cat_/*K*_M_ values were reduced for cefuroxime, cefoxitin and imipenem [86]. A detailed study of this position, involving replacement by all the possible amino acids, showed that various amino acids are tolerated at position 233, maintaining catalytic efficiencies and resistance towards different β-lactams, with the exception of imipenem [88]. Although IMP functionally accept replacements at this position, it is clear from its conservation that natural selection favors Asn. 

IMP-25 differs from IMP-6 by the G235S mutation at loop L10 (Figure 7 and Supplementary File 2), in close proximity to Asn233, which influences the substrate preferences (Figure 10). It is possible that Ser235 hydrogen bonds Asn233, altering the kinetic parameters for some substrates, as discussed by Oelschlaeger and coworkers [83]. At this position, other IMP variants present an Asp residue, which could also participate in a hydrogen bond with Asn233.

IMP-30 deviates from IMP-1 by a single mutation E59K (Figure 7 and Supplementary File 2), located at Loop-L3 [89]. This variant presents similar kinetics parameters to IMP-1, with the exception of ceftazidime, for which catalytic efficiency is six-fold higher. This translates to a two-fold increase on MIC relative to IMP-1 in *E. coli* cells. It is noteworthy that IMP-30 possesses the highest catalytic efficiency towards ceftazidime of all the described IMP variants [83,89] and also compared to the VIM variants [61], and to SPM-1 [90] and NDM-1 [14]. By means of computational docking and molecular dynamics simulation Oelschlaeger and coworkers inferred that the positively charged Lys residue at position 59 could stabilize an enzyme-ceftazidime complex during the catalytic cycle through an interaction with the negatively charged R1 group of ceftazidime [89].Various positions at Loop-L3 were also randomized to evaluate its role on substrate recognition. When ampicillin was used for the screening, most residues did not tolerate substitutions or only permitted a narrow variety of mutations. Instead, a non-wild type amino acid was prevalent in the selected variants at positions 61 and 62 (mutations V61L and N62P or N62A, respectively), suggesting that these positions may tune substrate preferences (Figure 9b) [44]. Interestingly, natural IMP-variants show Asn, Lys, Thr, and Ser, at position 62 (Figure 7 and Supplementary File 2). When other substrates were assayed, positions 61 and 67, at the base of this loop, accepted various residues in addition to the native Val, with a bias towards hydrophobic amino acids [73]. This observation is in line with the finding that several IMP alleles present Phe, Ala or Ile residues at position 67. The side chains of residues Val61 and Val67 contribute to formation of a hydrophobic pocket thought to bind the R1 substituent of substrates. Phe87 is also part of this cleft, and accepted a narrower variety of hydrophobic residues (Figure 9b). In coincidence, a few IMP variants present an Ile residue, but only two accept a Ser. The most stringent sequence requirements for this pocket are for ampicillin hydrolysis, suggesting the existence of an important interaction between the hydrophobic pocket in the enzyme and the benzyl group in the side-chain of ampicillin. Instead, for cefotaxime hydrolysis, a Gly residue was most common at position 87, probably because the removal of the Phe87 side chain leaves more space for the bulky R1 substituent of cefotaxime [73].

Trp64 is located at the tip of Loop-L3 between residues Gly63 and Gly65 (which do not tolerate changes). It has been proposed that this Trp residue plays an important role through its interaction with substrates [54]. Nevertheless, this position tolerates substitutions by other residues against all substrates tested, with preferences towards large, hydrophobic amino acids [73].

Other related positions essential for hydrolysis of one or more substrates, are Lys69 and Asp84 [73]. In the IMP-1 structure, Lys69 participates in an electrostatic interaction with Asp84, near the active site (Figure 9a). Both residues are critical for hydrolysis of cephalosporins but not for that of ampicillin or imipenem. Anyway, Asp84 is one of the most conserved residues in MBLs, adopting a disallowed conformation in the Ramachandran plots, thus, being essential for the overall MBL fold. Lys69 is also totally conserved on IMP variants. Table 2 presents a summary of the most important mutations discussed among the IMP variants that have an effect in the kinetic parameters of these enzymes. 

**Table 2:** Substrate profile of the different IMP (Imipenemase) variants and mutations, with respect to IMP-1, that were proposed as responsible for the altered substrate profiles.

Variant	Substrate Profile, Compared to IMP-1	Mutations, Compared to IMP-1
**IMP-6**	Lower catalytic efficiency for penicillins, ceftazidime, cephalotin, and imipenem Higher k_cat_ for meropenem	S262G
**IMP-25**	Lower catalytic efficiency for penicillins, ceftazidime, cephalotin, and imipenem Higher k_cat_ for meropenem (also higher than IMP-6)	S262G, G235S
**IMP-30**	Higher catalytic efficiency for ceftazidime	E59K

In summary, in the IMP variants the second-sphere residue at position 262 tunes substrate preferences, with a Ser residue conferring a broader substrate profile and a Gly at that position improving activity towards newer carbapenems but with a reduction in the profile of the class of substrates hydrolyzed. As seen for the VIM variants, mutations on residues at the base of Loop-L3 might fine tune substrate preferences. Positions 61 and 67 at the base of the loop accepted various residues instead of the native Val, with a bias towards hydrophobic residues. The hydrophobic pocket generated in the active site by the presence of a Phe residue at position 87 seems to favor interactions with some antibiotics that possess hydrophobic groups but to interfere with bulky R1 substituents in other β-lactams. Substrate binding relies on key interactions with the conserved residues Lys224 and Asn233 on Loop-L10. Position Lys224 is mostly intolerant to variations; whereas position 233 is more tolerant to randomization. However, an Asn at position 233 is critical for imipenem resistance, and indeed this is the amino acid that has been selected by natural evolution.

## 5. NDM Variants

NDM-1 was first identified in 2008, from *Klebsiella pneumonia* and *E. coli* strains isolated from a patient that acquired an infection in New Delhi, India [14]. The spread of NDM-positive bacteria has a complex epidemiology involving a variety of harboring species (principally *Klebsiella pneumonia* and *E. coli*) and inter-strain, inter-species, and inter-genus transmission of diverse plasmids containing *blaNDM* gene in more than 40 countries worldwide [91,92]. In fact, the gene spread has been the dominant dissemination mechanism [91].

Many of the initial NDM isolates indicated a link to the Indian subcontinent [93]. In the UK, positive isolates for NDM-1 have been documented since 2008, all of them with epidemiological link to India [93,94]. In addition, reports from different countries worldwide (Far and Middle East, USA and Canada and many countries in Europe) have documented NDM-1 presence with links to India [91]. However, these links were not always related to hospitalization, since some isolates were from community-associated infections, in patients that only had a history of travel to India. In addition, while numerous reports described the occurrence of NDM-1 positive bacteria in Indian hospitals [91], a measurement of the prevalence of the NDM-1 gene in drinking water and seepage samples in New Delhi found that it was carried by various strains in a worrisome frequency [95]. This indicates that NDM spread has passed the hospital walls and that environmental transmission is occurring [18,91]. In addition, many patients colonized with NDM-1 producers originated from the Balkan states, pointing it as a second reservoir [96,97]. Apart from that, many reports informed the presence of isolates from different countries with no history of travel to India, indicating the possibility of transmission by asymptomatic patients and local spread following importation [91].

NDM-1 gene is carried by diverse plasmids that also harbor multiple resistance genes associated with carbapenemases, cephalosporinases, macrolide resistance, rifampin, and sulfamethoxazole resistance, a combination that makes these strains multidrug resistant [5,95].

NDM-1 shows a broad susbtrate profile, being able to hydrolyze penicillins, carbapenems, and cephalosporins (with lower activity towards cefoxitin and ceftazidime) [14]. The NDM variants that have been isolated to date are less diversified than VIM and IMP variants, with only one or two mutations differentiating them. NDM-4, NDM-5, NDM-7, and NDM-8 present the M154L mutation in Loop L8 (position 150a according to BBL numbering) [98,99,100,101]. Biochemical characterization of NDM-4 showed slight increases in activity towards cephalosporins (cefatzidime, cephalotin, and cefotaxime) and carbapenems (imipenem and meropenem) with associated minor increments on MIC for carbapenems [98]. MIC assays with NDM-5 showed increased values compared to NDM-1 for ceftazidime, cefotaxime and cefpirome [99]. There was also an increase on the carbapenems’ MICs, but only when the natural promoter was employed for expression in *E. coli*. The Met154 side chain interacts with the backbone of Loop-L7, which contains three zinc ligands (His116, His118, and Asp120), so it is likely that this mutation impacts on the metal site structure. This is a second sphere interaction that deserves further exploration.

Thus far, 11 NDM variants have been isolated, sequenced and deposited [21]. Taking into account that for the VIM and IMP variants, 40 and 44 sequences have been isolated in a time span of 15 and 26 years respectively, and the rapid spread of NDM genes, we would expect more NDM variants to be discovered in the next years.

## 6. *In Vitro* Directed Evolution Performed on MBLs

An *in vitro* evolution experiment was performed with IMP-1 enzyme to test its potentiality to improve resistance towards imipenem [102]. Hall created a mutant library by amplification with the highly error prone polymerase Mutazyme and the genes were selected for their capacity to confer resistance to imipenem to *E. coli* cells harboring them. Initial MIC conferred by IMP-1 was 2 µg/mL and the experiment failed to detect any variant with increased MIC. There was an average of 1.2 mutations per gene, which means that single and double mutants were screened [102]. Though the author claims that IMP-1 cannot further increase its activity towards imipenem, some cautions should be taken. *E. coli* is particularly permeable to imipenem and as a result MBLs fail to confer the high levels of resistance observed in other gram-negative bacteria which have less permeable outer membrane barriers [103]. Then, it is possible that the system employed for detection was not sensible enough to detect improved variants. Additionally, the possibility remains that improved variants would arise through the accumulation of two or more mutations after more rounds of mutagenesis and selection. 

**Figure 11:**
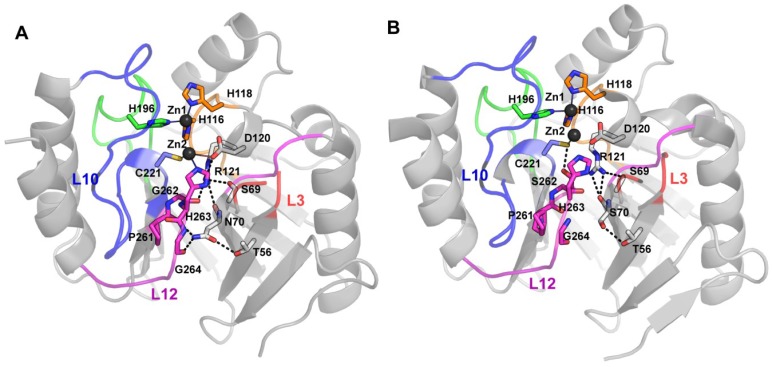
Hydrogen bond rearrangements in the vicinity of the active of the BcII evolved mutant. (**A**) BcII structure (PDB 1BC2); (**B**) BcII evolved mutant structure (PDB 3FCZ). The residues implicated in the hydrogen bond network that is affected by mutations N70S and G262S in the evolved mutant are shown together with the active site residues. The mutation G262S gives rise to a new interaction between side chains of Cys221 and Ser262 that affects the position of the Zn(II) ion at the Zn2 site. Interactions involving Asn70 side chain that connect Loop-12 and Loop-L3 are lost when this position is mutated to Ser. Residues are shown as sticks, relevant hydrogen bond interactions are shown as dashed lines and Zn(II) ions are shown as dark gray spheres.

In contrast, a successful *in vitro* evolution experiment was carried out with the B1 enzyme BcII by us [28]. This enzyme was employed as a model system to test the potentiality of MBLs to evolve their activity towards poor substrates, that is to say their propensity to accumulate mutations that would further broaden their substrate spectrum [28]. The DNA Shuffling technique was used to generate a library of random mutants of BcII, which were selected based on their ability to confer resistance to cephalexin to *E. coli* cells. After four rounds of mutagenesis and selection, clones with 32-fold increase on MIC were isolated. One of these variants presented four mutations (N70S, V112A, L250S, and G262S) and showed an increased hydrolytic efficiency towards cephalexin, principally due to the G262S mutation. G262S and N70S are second sphere mutations. G262S influences the position of the metal ion in the Zn2 site, thus affecting catalysis, while N70S affects the arrangement of hydrogen bonds involving Loops L3, L10, and L12, at the vicinity of the active site (Figure 11) [29]. The variant preserved high catalytic efficiency towards other substrates, which means that MBLs can expand their substrate profile by means of second sphere rearrangements. 

The mutation G262S is the same that differentiates IMP-1 (Ser at position 262) from IMP-6 (Gly at position 262), which as described on the previous section broadens the substrate profile of IMP-1. Then, it reinforces the conclusion that this position can tune substrate preferences and also validates directed molecular evolution approaches to predict possible variations in Nature. In the context of the hypothesis that a few thousand scaffolds have been profited throughout evolution to catalyze a wide spectrum of reactions or to fulfill a wide range of functions, it is interesting to explore the capacity to introduce new activities to an evolutionarily conserved protein scaffold through *in vitro* evolution experiments [104,105]. Such an approach was applied by Kim and coworkers to engineer lactamase activity on a member of the metallo-β-lactamase superfamily, the enzyme glyoxalase II, which catalyzes the hydrolysis of the thiolester bond of S-D-lactoylglutathione (a critical step in the conversion of cytotoxic 2-oxoaldehyde into 2-hydroxycarboxylic acids, that occurs in the cytoplasm) [106]. In order to succeed in this endeavor, it was necessary to incorporate and adjust functional elements by rational design, followed by conventional directed evolution methods. The evolved version of the engineered enzyme presented a *k*_cat_/*K*_M_ of 1.8 × 10^2^ M^−1^ s^−1^ and improved resistance towards cefotaxime by a factor of 100 with respect to the non-evolved one [106]. A subsequent work created libraries from this evolved variant by codon randomization of positions predicted to be involved in substrate binding -a “Focused DNA sequence library”- and by error prone PCR [107]. This study showed that it was much easier to increment the activity towards Penicillin G than towards cephalosporins. When selected variants from the “Focused DNA sequence library” were subjected to a round of error prone PCR, the new library performed better towards all the tested substrates, penicillins and cephalosporins. A multivariate analysis of resistance profiles towards the different substrates showed that the evolved variants are “generalists”, and then supports the conclusion that new activities can evolve in a general scaffold and that MBLs can evolve simultaneously improving and/or maintaining activities towards various β-lactam substrates [107].

## 7. Second Sphere Residues Determining Substrate Preferences and Zn(II) Binding

**Figure 12:**
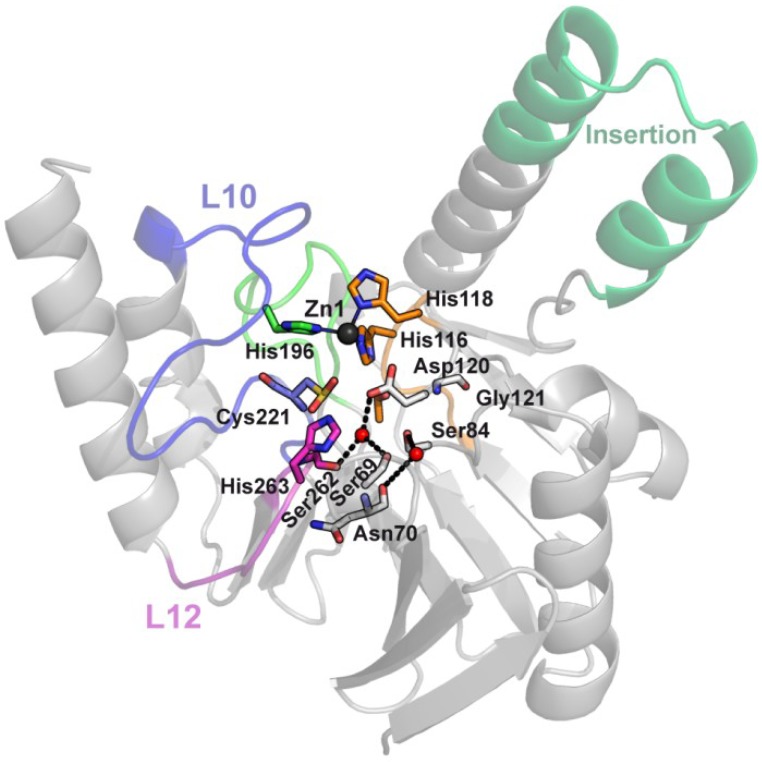
SPM-1, a B1 enzyme with unique structural and second sphere features. SPM-1 crystallographic structure (PDB 2FHX) shows how a central insertion accommodates in an extended α-helix, instead of the L8 loop found in the other B1 enzymes. Two atypical second sphere residues in SPM-1, S84, and G121, which replace the conserved D84/R121 couple, disrupt a B1 conserved second sphere network. Residues are shown as sticks, Zn(II) ions as dark gray spheres and water molecules as red spheres.

SPM-1 is a MBL produced only by *P. aeruginosa*, which makes it an ideal system to study host-specific evolutionary constraints. It is notable that, in contrast to other reported MBLs, there is only one known SPM-1 allele to date, which is only present in *P. aeruginosa* strains. The structure of SPM-1 reported by Spencer and coworkers reveals unique features among pathogen-associated MBLs. First, it lacks the L3 loop and accommodates a central insertion in an extended helical interdomain region (Figure 12) [56]. Second, B1 MBLs share a hydrogen bond network below the active site that implicates second sphere residues. This network is disrupted by the presence of two atypical second sphere residues in SPM-1, S84, and G121 (Figure 12), which replace the D84/R121 couple. As mentioned earlier, Asp84 is one of the most conserved residues in MBLs, adopting a disallowed conformation in the Ramachandran plots, thus, being essential for the overall MBL fold, while Arg121 is only partially conserved: it is replaced by S121 in IMP variants, C121 in CcrA and K121 in NDM-1. Because the side-chain charge is conserved on NDM-1 at this position, K121 in NDM-1 plays an equivalent role of R121 on the hydrogen bond network. The role of these positions was examined by codon randomization and selection in *P. aeruginosa* against various β-lactams [108]. Wild type clones (with residues S84 and G121) and mutants G121A, S84N, and S84N/G121S were selected against all tested antibiotics. On the other hand, some particular substitutions were isolated depending on the screening antibiotic, implying that positions 84 and 121 modulate the substrate profile of the enzyme. The B1 typical residue was accepted at position 84 (mutation S84D) but that was not the case for position 121 (mutation G121R was not found). For cefepime and imipenem, the wild type variant and G121A mutant show the highest specific activities. Instead, many single and double mutants in positions 84 and 121 out-performed wild type SPM-1 when evaluated against several antibiotics to which *P. aeruginosa* is intrinsically resistant. This fact demonstrates that the atypical S84/G121 combination present in SPM-1 has been fixed to provide resistance to anti-pseudomonal drugs, while sacrificing the catalytic efficiency towards other antibiotics, revealing that second sphere residues modulate the substrate specificity of MBLs according to the requirements of the bacterial host.

The effect of second sphere residue mutations does not exhaust on catalytic efficiency. Our group has shown that the Zn(II) binding affinity of MBLs influences MIC values [32]. More precisely, mutation of one of the metal ligands (C221D) at the active site of BcII maintained catalytic efficiency but decreased Zn(II) binding affinity and hence resulted in a drastic reduction in the MIC values. Unpublished results from our group also showed that second sphere mutations on the evolved variant of BcII obtained by directed molecular evolution (the variant carrying the mutations N70S, V112A, L250S and G262S) modulate the enzyme’s Zn(II) binding affinity. In addition, our study on the above-mentioned atypical residues S84 and G121 found in the second sphere of SPM-1, revealed that this combination optimizes the zinc binding affinity of SPM-1 in the bacterial periplasm, rendering *P. aeruginosa* resistant to β-lactam antibiotics under zinc-limiting conditions [108]. In fact, this study indicated that the variant with the highest specific activity in metal-rich media (G121A), which out-competed wild type SPM-1 under those conditions, was extremely sensitive to metal deprivation. Native SPM-1, instead, is able to confer resistance under conditions of Zn(II) deficiency, which suggests that in *P. aeruginosa* evolutionary pressure has been exerted to select MBL variants capable of providing resistance in environments with low Zn(II) concentrations. This is particularly important during bacterial infection, since mammalian hosts elicit a defense strategy, known as nutritional immunity, to limit Zn(II) availability [109,110]. In summary, these studies clearly indicate that the affinity for the metal ion is also a trait that is subject to optimization during evolution of MBLs.

## 8. Conclusions

Overall, we have shown the complementarity of different approaches to tackle the controversial issue of antibiotic resistance, in this particular case, focusing on MBLs. On one hand, the analysis of mutants present in Nature is of relevance when biochemical, structural and microbiological studies are analyzed altogether. Otherwise, spare reports do not allow a precise assessment of the adaptation mechanism of these enzymes. This approach is quite complementary to the generation of mutants *in vitro* coupled to a high-throughput analysis and selection, which mimic natural selection, and indeed, has proven useful to anticipate mutations in the natural environment. 

A comparison of the available mutants in the numerous VIM and IMP families of MBLs surprisingly reveals that these groups have evolved their substrate specificities by different mechanisms. VIM enzymes accumulate mutations in the loops flanking the active site, mostly affecting the electrostatic interaction with the substrate carboxylate or loop flexibility. The same mechanism is observed for some IMP variants. In addition, substrate preferences among IMP variants are tuned by second sphere mutations. In this regard, directed evolution approaches have also provided improved MBLs by accumulation of mutations in the second sphere in similar positions, suggesting the existence of possible hot spots for adaptive mutagenesis. Finally, codon randomization is equally important to explore new hypothesis on the role of second sphere residues, and has been able to provide information on how SPM-1 has adapted the substrate profile according to the needs of one bacterial host (*Pseudomonas aeruginosa*). Instead, VIM and IMP enzymes show many different alleles which have clearly been selected in different clinical strains under distinct environments. The number of NDM variants is still limited but, considering the little time elapsed since its first report, may present a different evolutionary scenario. More inter- and multidisciplinary studies are needed in this field, since inhibitor design cannot overlook the rapid evolving features of this family of enzymes. 

## Supplementary Files

Supplementary File 1

Supplementary File 2
