# Supplementary material for: Evolution of Metallo-β-lactamases: Trends Revealed by Natural Diversity and in vitro Evolution
Source: Antibiotics (Basel). 2014 Jul 1;3(3):285–316. doi: 10.3390/antibiotics3030285 (PMC4212336; doi:10.3390/antibiotics3030285)
Supplement: Supplementary File 1 [file antibiotics-03-00285-s001.pdf]

VIM variants protein sequence alignment- BBL numbering

	0	1	2	3	4	5	6	7	8	9	10	11	12	13	14	15	16	17	18	19	20	21	22	23	24	25	26	27	28	29	30	31	32	33	34	35
VIM-1	M	L	K	V	I	S	S	L	L	V	Y	M	T	A	S	V	M	A	V	A	S	P	L	A	H	S	G	E	P	S	G	E	Y	P	T	V
VIM-2	M	F	K	L	L	S	K	L	L	V	Y	L	T	A	S	I	M	A	I	A	S	P	L	A	F	S	V	D	S	S	G	E	Y	P	T	V
VIM-3	M	F	K	L	L	S	K	L	L	V	Y	L	T	A	S	I	M	A	I	A	S	P	L	A	F	S	V	D	S	S	G	E	Y	P	T	V
VIM-4	M	L	K	V	I	S	S	L	L	V	Y	M	T	A	S	V	M	A	V	A	S	P	L	A	H	S	G	E	P	S	G	E	Y	P	T	V
VIM-5	M	L	K	V	I	S	S	L	L	V	Y	M	T	A	S	V	M	A	V	A	S	P	L	A	H	S	G	E	P	S	G	E	Y	P	T	V
VIM-6	M	F	K	L	L	S	K	L	L	V	Y	L	T	A	S	I	M	A	I	A	S	P	L	A	F	S	V	D	S	S	G	E	Y	P	T	V
VIM-7	M	F	Q	I	R	S	F	L	V	G	I	S	A	F	V	-	M	A	V	L	G	S	A	A	Y	S	A	Q	P	G	G	E	Y	P	T	V
VIM-8	M	F	K	L	L	S	K	L	L	V	Y	L	T	A	S	I	M	A	I	A	S	P	L	A	F	S	V	D	S	S	G	E	Y	P	T	V
VIM-9	M	F	K	L	L	S	K	L	L	V	Y	L	T	A	S	I	M	A	I	A	S	P	L	A	F	S	V	D	S	S	G	E	Y	P	T	V
VIM-10	M	F	K	L	L	S	K	L	L	V	Y	L	T	A	S	I	M	A	I	A	S	P	L	A	F	S	V	D	S	S	G	E	Y	P	T	V
VIM-11	M	F	K	L	L	S	K	L	L	V	Y	L	T	A	S	I	M	A	I	A	S	P	L	A	F	S	V	D	S	S	G	E	Y	P	T	V
VIM-12	M	L	K	V	I	S	S	L	L	V	Y	M	T	A	S	V	M	A	V	A	S	P	L	A	H	S	G	E	P	S	G	E	Y	P	T	V
VIM-13	M	L	K	V	I	S	S	L	L	F	Y	M	T	A	S	L	M	A	V	A	S	P	L	A	H	S	G	E	S	R	G	E	Y	P	T	V
VIM-14	M	L	K	V	I	S	S	L	L	V	Y	M	T	A	S	V	M	A	V	A	S	P	L	A	H	S	G	E	P	S	S	E	Y	P	T	V
VIM-15	M	F	K	L	L	S	K	L	L	V	Y	L	T	A	S	I	M	A	I	A	S	P	L	A	F	S	V	D	S	S	G	E	Y	P	T	V
VIM-16	M	F	K	L	L	S	K	L	L	V	Y	L	T	A	S	I	M	A	I	A	S	P	L	A	F	S	V	D	S	S	G	E	Y	P	T	V
VIM-17	M	F	K	L	L	S	K	L	L	V	Y	L	T	A	S	M	M	A	I	A	S	P	L	A	F	S	V	D	S	S	G	E	Y	P	T	V
VIM-18	M	F	K	L	L	S	K	L	L	V	Y	L	T	A	S	I	M	A	I	A	S	P	L	A	F	S	V	D	S	S	G	E	Y	P	T	V
VIM-19	M	L	K	V	I	S	S	L	L	V	Y	M	T	A	S	V	M	A	V	A	S	P	L	A	H	S	G	E	P	S	G	E	Y	P	T	V
VIM-23	M	F	K	L	L	S	K	L	L	V	Y	L	T	A	S	I	M	A	I	A	S	P	L	A	F	S	V	D	S	S	G	E	Y	P	T	V
VIM-24	M	F	K	L	L	S	K	L	L	V	Y	L	T	A	S	I	M	A	I	A	S	P	L	A	F	S	V	D	S	S	G	E	Y	P	T	V
VIM-25	M	L	K	V	I	S	S	L	L	V	Y	M	T	A	S	V	M	A	V	A	S	P	L	A	H	S	G	E	P	S	G	E	Y	P	T	V
VIM-26	M	L	K	V	I	S	S	L	L	V	Y	M	T	A	S	V	M	A	V	A	S	P	L	A	H	S	G	E	P	S	G	E	Y	P	T	V
VIM-27	M	L	K	V	I	S	S	L	L	V	Y	M	T	A	S	V	M	A	V	A	S	P	L	A	H	S	G	E	P	S	G	E	Y	P	T	V
VIM-28	M	L	K	V	I	S	S	L	L	V	Y	M	T	A	S	V	M	A	V	A	S	P	L	A	H	S	G	E	P	S	G	E	Y	P	T	V
VIM-29	M	L	K	V	I	S	S	L	L	V	Y	M	T	A	S	V	M	A	V	A	S	P	L	A	H	S	G	E	P	S	G	E	Y	P	T	V
VIM-30	M	F	K	L	L	S	K	L	L	V	Y	L	T	A	S	I	M	A	I	A	S	P	L	A	F	S	V	D	S	S	G	E	Y	P	T	V
VIM-31	M	F	K	L	L	S	K	L	L	V	Y	L	T	A	S	I	M	A	I	A	S	P	L	A	F	S	V	D	S	S	G	E	Y	P	T	V
VIM-32	M	L	K	V	I	S	S	L	L	V	Y	M	T	A	S	V	M	A	V	A	S	P	L	A	H	S	G	E	P	S	G	E	Y	P	T	V
VIM-33	M	L	K	V	I	S	S	L	L	V	Y	M	T	A	S	V	M	A	V	A	S	P	L	A	H	S	G	E	P	S	G	E	Y	P	T	V
VIM-34	M	L	K	V	I	S	S	L	L	V	Y	M	T	A	S	V	M	A	V	A	S	P	L	A	H	S	G	E	P	S	G	E	Y	P	T	V
VIM-35	M	L	K	V	I	S	S	L	L	V	Y	M	T	A	S	V	M	A	V	A	S	P	L	A	H	S	G	E	P	S	G	E	Y	P	T	V
VIM-36	M	F	K	L	L	S	K	L	L	V	Y	L	T	A	S	I	M	A	I	A	S	P	L	A	F	S	V	D	S	S	G	E	Y	P	T	V
VIM-37	M	L	K	V	I	S	S	L	L	V	Y	M	T	A	S	V	M	A	V	A	S	P	L	A	H	S	G	E	P	S	G	E	Y	P	T	V
VIM-38	M	L	K	V	I	S	S	L	L	V	Y	L	T	A	S	V	M	A	V	A	S	P	L	A	H	S	G	E	P	S	G	E	Y	P	T	V

Gray: Signal peptide; Red: Zn(II) ligands; Green: positions related to substrate preferences

VIM variants protein sequence alignment- BBL numbering

	36	37	38	39	40	41	42	43	44	45	46	47	48	49	50	51	52	53	54	55	56	57	58	59	60	61	62	63	65	66	67	68	69	70	71	72
VIM-1	N	E	I	P	V	G	E	V	R	L	Y	Q	I	A	D	G	V	W	S	H	I	A	T	Q	S	F	D	G	A	V	Y	P	S	N	G	L
VIM-2	S	E	I	P	V	G	E	V	R	L	Y	Q	I	A	D	G	V	W	S	H	I	A	T	Q	S	F	D	G	A	V	Y	P	S	N	G	L
VIM-3	S	E	I	P	V	G	E	V	R	L	Y	Q	I	A	D	G	V	W	S	H	I	A	T	K	S	F	D	G	A	V	Y	P	S	N	G	L
VIM-4	N	E	I	P	V	G	E	V	R	L	Y	Q	I	A	D	G	V	W	S	H	I	A	T	Q	S	F	D	G	A	V	Y	P	S	N	G	L
VIM-5	N	E	I	P	V	G	E	V	R	L	Y	Q	I	A	D	G	V	W	S	H	I	A	T	Q	S	F	D	G	A	V	Y	P	S	N	G	L
VIM-6	S	E	I	P	V	G	E	V	R	L	Y	Q	I	A	D	G	V	W	S	H	I	A	T	R	S	F	D	G	A	V	Y	P	S	N	G	L
VIM-7	D	D	I	P	V	G	E	V	R	L	Y	K	I	G	D	G	V	W	S	H	I	A	T	Q	K	L	G	D	T	V	Y	S	S	N	G	L
VIM-8	S	E	I	P	V	G	E	V	R	L	Y	Q	I	A	D	G	V	W	S	H	I	A	T	Q	S	F	D	G	A	V	Y	P	S	N	G	L
VIM-9	S	E	I	P	V	G	E	V	R	L	Y	Q	I	A	D	G	V	W	S	H	I	A	T	Q	S	F	D	G	A	V	Y	P	S	N	G	L
VIM-10	S	E	I	P	V	G	E	V	R	L	Y	Q	I	A	D	G	V	W	S	H	I	A	T	Q	S	F	D	G	A	V	Y	P	S	N	G	L
VIM-11	S	E	I	P	V	G	E	V	R	L	Y	Q	I	A	D	G	V	W	S	H	I	A	T	Q	S	F	D	G	A	V	Y	P	S	N	G	L
VIM-12	N	E	I	P	V	G	E	V	R	L	Y	Q	I	A	D	G	V	W	S	H	I	A	T	Q	S	F	D	G	A	V	Y	P	S	N	G	L
VIM-13	S	E	I	P	V	G	E	V	R	L	Y	Q	I	D	D	G	V	W	S	H	I	A	T	H	T	F	D	G	V	V	Y	P	S	N	G	L
VIM-14	N	E	I	P	V	G	E	V	R	L	Y	Q	I	A	D	G	V	W	S	H	I	A	T	Q	S	F	D	G	A	V	Y	P	S	N	G	L
VIM-15	S	E	I	P	V	G	E	V	R	L	Y	Q	I	A	D	G	V	W	S	H	I	A	T	Q	S	F	D	G	A	V	Y	P	S	N	G	L
VIM-16	S	E	I	P	V	G	E	V	R	L	Y	Q	I	A	D	G	V	W	L	H	I	A	T	Q	S	F	D	G	A	V	Y	P	S	N	G	L
VIM-17	S	E	I	P	V	G	E	V	R	L	Y	Q	I	A	D	G	V	W	S	H	I	A	T	Q	S	F	D	G	A	V	Y	P	S	N	G	L
VIM-18	S	E	I	P	V	G	E	V	R	L	Y	Q	I	A	D	G	V	W	S	H	I	A	T	R	S	F	D	G	A	V	Y	P	S	N	G	L
VIM-19	N	E	I	P	V	G	E	V	R	L	Y	Q	I	A	D	G	V	W	S	H	I	A	T	Q	S	F	D	G	A	V	Y	P	S	N	G	L
VIM-23	S	E	I	P	V	G	E	V	R	L	Y	Q	I	A	D	G	V	W	S	H	I	A	T	Q	S	F	D	G	A	V	Y	P	S	N	G	L
VIM-24	S	E	I	P	V	G	E	V	R	L	Y	Q	I	A	D	G	V	W	S	H	I	A	T	Q	S	F	D	G	A	V	Y	P	S	N	G	L
VIM-25	N	E	I	P	V	G	E	V	R	L	Y	Q	I	A	D	G	V	W	S	H	I	A	T	Q	S	F	D	G	A	V	Y	P	S	N	G	L
VIM-26	N	E	I	P	V	G	E	V	R	L	Y	Q	I	A	D	G	V	W	S	H	I	A	T	Q	S	F	D	G	A	V	Y	P	S	N	G	L
VIM-27	N	E	I	P	V	G	E	V	R	L	Y	Q	I	A	D	G	V	W	S	H	I	S	T	Q	S	F	D	G	A	V	Y	P	S	N	G	L
VIM-28	N	E	I	P	V	G	E	V	R	L	Y	Q	I	A	D	G	V	W	S	H	I	A	T	Q	S	F	D	G	A	V	Y	P	S	N	G	L
VIM-29	N	E	I	P	V	G	E	V	R	L	Y	Q	I	A	D	G	V	W	S	H	I	A	T	Q	S	F	D	G	A	V	Y	P	S	N	G	L
VIM-30	N	E	I	P	V	G	E	V	R	L	Y	Q	I	A	D	G	V	W	S	H	I	A	T	Q	S	F	D	G	A	V	Y	P	S	N	G	L
VIM-31	S	E	I	P	V	G	E	V	R	L	Y	Q	I	A	D	G	V	W	S	H	I	A	T	Q	S	F	D	G	A	V	Y	P	S	N	G	L
VIM-32	N	E	I	P	V	G	E	V	R	L	Y	Q	I	A	D	G	V	W	S	H	I	A	T	Q	S	F	D	G	A	V	Y	P	S	N	G	L
VIM-33	N	E	I	P	V	G	E	V	R	L	Y	Q	I	A	D	G	V	W	S	H	I	A	T	Q	S	F	D	G	A	V	Y	P	S	N	G	L
VIM-34	N	E	I	P	V	G	E	V	R	L	Y	Q	I	A	D	G	V	W	S	H	I	A	T	Q	S	F	D	G	A	V	Y	P	S	N	G	L
VIM-35	N	E	I	P	V	G	E	V	R	L	Y	Q	I	A	D	G	V	W	S	H	I	A	T	Q	S	F	D	G	A	V	Y	P	S	N	G	L
VIM-36	S	E	I	P	V	G	E	V	R	L	Y	Q	I	A	D	G	V	W	S	H	I	A	T	R	S	F	D	G	A	V	Y	P	S	N	G	L
VIM-37	N	E	I	P	V	G	E	V	R	L	Y	Q	I	A	D	G	V	W	S	H	I	S	T	Q	S	F	D	G	A	V	Y	P	S	N	G	L
VIM-38	N	E	I	P	V	G	E	V	R	L	Y	Q	I	A	D	G	V	W	S	H	I	A	T	Q	S	F	D	G	A	V	Y	P	S	N	G	L

Gray: Signal peptide; Red: Zn(II) ligands; Green: positions related to substrate preferences

VIM variants protein sequence alignment- BBL numbering

	73	74	75	76	77	78	79	80	81	82	83	84	85	86	87	88	89	90	91	92	93	94	95	96	97	98	99	100	101	102	103	104	107	108	109
VIM-1	I	V	R	D	G	D	E	L	L	L	I	D	T	A	W	G	A	K	N	T	A	A	L	L	A	E	I	E	K	Q	I	G	L	P	V
VIM-2	I	V	R	D	G	D	E	L	L	L	I	D	T	A	W	G	A	K	N	T	A	A	L	L	A	E	I	E	K	Q	I	G	L	P	V
VIM-3	I	V	R	D	G	D	E	L	L	L	I	D	T	A	W	G	A	K	N	T	A	A	L	L	A	E	I	E	K	Q	I	G	L	P	V
VIM-4	I	V	R	D	G	D	E	L	L	L	I	D	T	A	W	G	A	K	N	T	A	A	L	L	A	E	I	E	K	Q	I	G	L	P	V
VIM-5	I	V	R	D	G	D	E	L	L	L	I	D	T	A	W	G	A	K	N	T	A	A	L	L	A	E	I	E	K	Q	I	G	L	P	V
VIM-6	I	V	R	D	G	D	E	L	L	L	I	D	T	A	W	G	A	K	N	T	A	A	L	L	A	E	I	E	K	Q	I	G	L	P	V
VIM-7	I	V	R	D	A	D	E	L	L	L	I	D	T	A	W	G	A	K	N	T	V	A	L	L	A	E	I	E	K	Q	I	G	L	P	V
VIM-8	I	V	R	D	G	D	E	L	L	L	I	D	T	A	W	G	A	K	N	T	A	A	L	L	A	E	I	E	K	Q	I	G	L	P	V
VIM-9	I	V	R	D	G	D	E	L	L	L	I	D	T	A	W	G	A	K	N	T	A	A	L	L	A	E	I	E	K	Q	I	G	L	P	V
VIM-10	I	V	R	D	G	D	E	L	L	L	I	D	T	A	W	G	A	K	N	T	A	A	L	L	A	E	I	E	K	Q	I	G	L	P	V
VIM-11	I	V	R	D	G	D	E	L	L	L	I	D	T	A	W	G	A	K	N	T	A	A	L	L	A	E	I	E	K	Q	I	G	L	P	V
VIM-12	I	V	R	D	G	D	E	L	L	L	I	D	T	A	W	G	A	K	N	T	A	A	L	L	A	E	I	E	K	Q	I	G	L	P	V
VIM-13	I	V	R	D	G	D	E	L	L	L	I	D	T	A	W	G	T	K	N	T	V	A	L	L	A	E	I	E	K	Q	I	G	L	P	V
VIM-14	I	V	R	D	G	D	E	L	L	L	I	D	T	A	W	G	A	K	N	T	A	A	L	L	A	E	I	E	K	Q	I	G	L	P	V
VIM-15	I	V	R	D	G	D	E	L	L	L	I	D	T	A	W	G	A	K	N	T	A	A	L	L	A	E	I	E	K	Q	I	G	L	P	V
VIM-16	I	V	R	D	G	D	E	L	L	L	I	D	T	A	W	G	A	K	N	T	A	A	L	L	A	E	I	E	K	Q	I	G	L	P	V
VIM-17	I	V	R	D	G	D	E	L	L	L	I	D	T	A	W	G	A	K	N	T	A	A	L	L	A	E	I	E	K	Q	I	G	L	P	V
VIM-18	I	V	R	D	G	D	E	L	L	L	I	D	T	A	W	G	A	K	N	T	A	A	L	L	A	E	I	E	K	Q	I	G	L	P	V
VIM-19	I	V	R	D	G	D	E	L	L	L	I	D	T	A	W	G	A	K	N	T	A	A	L	L	A	E	I	E	K	Q	I	G	L	P	V
VIM-23	I	V	R	D	G	D	E	L	L	L	I	D	T	A	W	G	A	K	N	T	A	A	L	L	A	E	I	E	K	Q	I	G	L	P	V
VIM-24	I	V	R	D	G	D	E	L	L	L	I	D	T	A	W	G	A	K	N	T	A	A	L	L	A	E	I	E	K	Q	I	G	L	P	V
VIM-25	I	V	R	D	G	D	E	L	L	L	I	D	T	A	W	G	A	K	N	T	A	A	L	L	A	E	I	E	K	Q	I	G	L	P	V
VIM-26	I	V	R	D	G	D	E	L	L	L	I	D	T	A	W	G	A	K	N	T	A	A	L	L	A	E	I	E	K	Q	I	G	L	P	V
VIM-27	I	V	R	D	G	D	E	L	L	L	I	D	T	A	W	G	A	K	N	T	A	A	L	L	A	E	I	E	K	Q	I	G	L	P	V
VIM-28	I	V	R	D	G	D	E	L	L	L	I	D	T	A	W	G	A	K	N	T	A	A	L	L	A	E	I	E	K	Q	I	G	L	P	V
VIM-29	I	V	R	D	G	D	E	L	L	L	I	D	T	A	W	G	A	K	N	T	A	A	L	L	A	E	I	E	K	Q	I	G	L	P	V
VIM-30	I	V	R	D	G	D	E	L	L	L	I	D	T	A	W	G	A	K	N	T	A	A	L	L	A	E	I	E	K	Q	I	G	L	P	V
VIM-31	I	V	R	D	G	D	E	L	L	L	I	D	T	A	W	G	A	K	N	T	A	A	L	L	A	E	I	E	K	Q	I	G	L	P	V
VIM-32	I	V	R	D	G	D	E	L	L	L	I	D	T	A	W	G	A	K	N	T	A	A	L	L	A	E	I	E	K	Q	I	G	L	P	V
VIM-33	I	V	R	D	G	D	E	L	L	L	I	D	T	A	W	G	A	K	N	T	A	A	L	L	A	E	I	E	K	Q	I	G	L	P	V
VIM-34	I	V	R	D	G	D	E	L	L	L	I	D	T	A	W	G	A	K	N	T	A	A	L	L	A	E	I	E	K	Q	I	G	L	P	V
VIM-35	I	V	R	D	G	D	E	L	L	L	I	D	T	A	W	G	A	K	N	T	A	A	L	L	A	E	I	E	K	Q	I	G	L	P	V
VIM-36	I	V	R	D	G	D	E	L	L	L	I	D	T	A	W	G	A	K	N	T	A	A	L	L	A	E	I	E	K	Q	I	G	L	P	V
VIM-37	I	V	R	D	G	D	E	L	L	L	I	D	T	A	W	G	A	K	N	T	A	A	L	L	A	E	I	E	K	Q	I	G	L	P	V
VIM-38	I	V	R	D	G	D	E	L	L	L	I	D	T	A	W	G	A	K	N	T	A	A	L	L	A	E	I	E	K	Q	I	G	L	P	V

Gray: Signal peptide; Red: Zn(II) ligands; Green: positions related to substrate preferences

VIM variants protein sequence alignment- BBL numbering

	110	111	112	113	114	115	**116**	117	**118**	119	**120**	121	122	123	124	125	126	127	128	129	130	131	133	134	135	136	137	138	139	140	141	142	143	144	145	146
VIM-1	T	R	A	V	S	T	**H**	F	**H**	D	**D**	R	V	G	G	V	D	V	L	R	A	A	G	V	A	T	Y	A	S	P	S	T	R	R	L	A
VIM-2	T	R	A	V	S	T	**H**	F	**H**	D	**D**	R	V	G	G	V	D	V	L	R	A	A	G	V	A	T	Y	A	S	P	S	T	R	R	L	A
VIM-3	T	R	A	V	S	T	**H**	F	**H**	D	**D**	R	V	G	G	V	D	V	L	R	A	A	G	V	A	T	Y	A	S	P	S	T	R	R	L	A
VIM-4	T	R	A	V	S	T	**H**	F	**H**	D	**D**	R	V	G	G	V	D	V	L	R	A	A	G	V	A	T	Y	A	S	P	S	T	R	R	L	A
VIM-5	T	R	A	V	S	T	**H**	F	**H**	D	**D**	R	V	G	G	V	D	V	L	R	K	A	G	V	A	T	Y	A	S	P	S	T	R	R	L	A
VIM-6	T	R	A	V	S	T	**H**	F	**H**	D	**D**	R	V	G	G	V	D	V	L	R	A	A	G	V	A	T	Y	A	S	P	S	T	R	R	L	A
VIM-7	T	R	S	I	S	T	**H**	F	**H**	D	**D**	R	V	G	G	V	D	V	L	R	A	A	G	V	A	T	Y	T	S	P	L	T	R	Q	L	A
VIM-8	T	R	A	V	S	T	**H**	F	**H**	D	**D**	R	V	G	G	V	D	V	L	R	A	A	G	V	A	T	Y	A	S	P	S	A	R	R	L	A
VIM-9	T	R	A	V	S	T	**H**	F	**H**	D	**D**	R	V	G	G	V	D	V	L	R	A	A	G	V	A	T	Y	A	S	P	S	I	R	R	L	A
VIM-10	T	R	A	V	S	T	**H**	F	**H**	D	**D**	R	V	G	G	V	D	V	L	R	A	A	G	V	A	T	Y	A	S	P	S	T	R	R	L	A
VIM-11	T	R	A	V	S	T	**H**	F	**H**	D	**D**	R	V	G	G	V	D	V	L	R	A	A	G	V	A	T	Y	A	S	P	S	T	R	R	L	A
VIM-12	T	R	A	V	S	T	**H**	F	**H**	D	**D**	R	V	G	G	V	D	V	L	R	A	A	G	V	A	T	Y	A	S	P	S	T	R	R	L	A
VIM-13	T	R	S	V	S	T	**H**	F	**H**	D	**D**	R	V	G	G	V	D	A	L	R	A	A	G	V	A	T	Y	A	S	P	S	T	R	R	L	A
VIM-14	T	R	A	V	S	T	**H**	F	**H**	D	**D**	R	V	G	G	V	D	V	L	R	A	A	G	V	A	T	Y	A	S	P	S	T	R	R	L	A
VIM-15	T	R	A	V	S	T	**H**	F	**H**	D	**D**	R	V	G	G	V	D	V	L	R	A	A	G	V	A	T	Y	A	S	P	S	T	R	R	L	A
VIM-16	T	R	A	V	S	T	**H**	F	**H**	D	**D**	R	V	G	G	V	D	V	L	R	A	A	G	V	A	T	Y	A	S	P	S	T	R	R	L	A
VIM-17	T	R	A	V	S	T	**H**	F	**H**	D	**D**	R	V	G	G	V	D	V	L	R	A	A	G	V	A	T	Y	A	S	P	S	T	R	R	L	A
VIM-18	T	R	A	V	S	T	**H**	F	**H**	D	**D**	R	V	G	G	V	D	V	L	R	A	A	G	V	A	T	Y	A	S	P	S	T	R	R	L	A
VIM-19	T	R	A	V	S	T	**H**	F	**H**	D	**D**	R	V	G	G	V	D	V	L	R	A	A	G	V	A	T	Y	A	S	P	S	T	R	R	L	A
VIM-23	T	R	A	V	S	T	**H**	F	**H**	D	**D**	R	V	G	G	V	D	V	L	R	A	A	G	V	A	T	Y	A	S	P	S	T	R	R	L	A
VIM-24	T	R	A	V	S	T	**H**	F	**H**	D	**D**	R	V	G	G	V	D	V	L	R	A	A	G	V	A	T	Y	A	S	P	S	T	R	R	L	A
VIM-25	T	R	A	V	S	T	**H**	F	**H**	D	**D**	R	V	G	G	V	D	V	L	R	K	A	G	V	A	T	Y	A	S	P	S	T	R	R	L	A
VIM-26	T	R	A	V	S	T	**H**	F	**H**	D	**D**	R	V	G	G	V	D	V	L	R	A	A	G	V	A	T	Y	A	S	P	S	T	R	R	L	A
VIM-27	T	R	A	V	S	T	**H**	F	**H**	D	**D**	R	V	G	G	V	D	V	L	R	A	A	G	V	A	T	Y	A	S	P	S	T	R	R	L	A
VIM-28	T	R	A	V	S	T	**H**	F	**H**	D	**D**	R	V	G	G	V	D	V	L	R	A	A	G	V	A	T	Y	A	S	P	S	T	R	R	L	A
VIM-29	T	R	A	V	S	T	**H**	F	**H**	D	**D**	R	V	G	G	V	D	V	L	R	A	A	G	V	A	T	Y	A	S	P	S	T	R	R	L	A
VIM-30	T	R	A	V	S	T	**H**	F	**H**	D	**D**	R	V	G	G	V	D	V	L	R	A	A	G	V	A	T	Y	A	S	P	S	T	R	R	L	A
VIM-31	T	R	A	V	S	T	**H**	F	**H**	D	**D**	R	V	G	G	V	D	V	L	R	A	A	G	V	A	T	Y	A	S	P	S	T	R	R	L	A
VIM-32	T	R	A	V	S	T	**H**	F	**H**	D	**D**	R	V	G	G	V	D	V	L	R	A	A	G	V	A	T	Y	A	S	P	S	T	R	R	L	A
VIM-33	T	R	A	V	S	T	**H**	F	**H**	D	**D**	R	V	G	G	V	D	V	L	R	A	A	G	V	A	T	Y	A	S	P	S	T	R	R	L	A
VIM-34	T	R	A	I	S	T	**H**	F	**H**	D	**D**	R	V	G	G	V	D	V	L	R	A	A	G	V	A	T	Y	A	S	P	S	T	R	R	L	A
VIM-35	T	R	A	V	S	T	**H**	F	**H**	D	**D**	R	V	G	G	V	D	V	L	R	A	A	G	V	A	T	Y	A	S	P	S	T	R	R	L	A
VIM-36	T	R	A	V	S	T	**H**	F	**H**	D	**D**	R	V	G	G	V	D	V	L	R	A	A	G	V	A	T	Y	A	S	P	S	T	R	R	L	A
VIM-37	T	R	A	V	S	T	**H**	F	**H**	D	**D**	R	V	G	G	V	D	V	L	R	A	A	G	V	A	T	Y	A	S	P	S	T	R	R	L	A
VIM-38	T	R	A	V	S	T	**H**	F	**H**	D	**D**	R	V	G	G	V	D	V	L	R	K	A	G	V	A	T	Y	A	S	P	S	T	R	R	L	A

Gray: Signal peptide; Red: Zn(II) ligands; Green: positions related to substrate preferences

VIM variants protein sequence alignment- BBL numbering

	147	148	149	150	a	162	163	164	165	166	167	168	169	170	171	172	173	174	175	176	177	178	179	180	181	182	183	184	185	190	191	192	193	194	195	**196**
VIM-1	E	A	E	G	N	E	I	P	T	H	S	L	E	G	L	S	S	S	G	D	A	V	R	F	G	P	V	E	L	F	Y	P	G	A	A	**H**
VIM-2	E	V	E	G	N	E	I	P	T	H	S	L	E	G	L	S	S	S	G	D	A	V	R	F	G	P	V	E	L	F	Y	P	G	A	A	**H**
VIM-3	E	V	E	G	S	E	I	P	T	H	S	L	E	G	L	S	S	S	G	D	A	V	R	F	G	P	V	E	L	F	Y	P	G	A	A	**H**
VIM-4	E	A	E	G	N	E	I	P	T	H	S	L	E	G	L	S	S	S	G	D	A	V	R	F	G	P	V	E	L	F	Y	P	G	A	A	**H**
VIM-5	E	A	E	G	N	E	I	P	T	H	S	L	E	G	L	S	S	S	G	D	A	V	R	F	G	P	V	E	L	F	Y	P	G	A	A	**H**
VIM-6	E	V	E	G	S	E	I	P	T	H	S	L	E	G	L	S	S	S	G	D	A	V	R	F	G	P	V	E	L	F	Y	P	G	A	A	**H**
VIM-7	E	A	A	G	N	E	V	P	A	H	S	L	K	A	L	S	S	S	G	D	V	V	R	F	G	P	V	E	V	F	Y	P	G	A	A	**H**
VIM-8	E	V	E	G	N	E	I	P	T	H	S	L	E	G	L	S	S	S	G	D	A	V	R	F	G	P	V	E	L	F	Y	P	G	A	A	**H**
VIM-9	E	V	E	G	N	E	I	P	T	H	S	L	E	G	L	S	S	S	G	D	A	V	R	F	G	P	V	E	L	F	Y	P	G	A	A	**H**
VIM-10	E	V	E	G	N	E	I	P	T	H	S	L	E	G	L	S	S	S	G	D	A	V	R	F	G	P	V	E	L	F	Y	P	G	A	A	**H**
VIM-11	E	V	E	G	S	E	I	P	T	H	S	L	E	G	L	S	S	S	G	D	A	V	R	F	G	P	V	E	L	F	Y	P	G	A	A	**H**
VIM-12	E	A	E	G	N	E	I	P	T	H	S	L	E	G	L	S	S	S	G	D	A	V	R	F	G	P	V	E	L	F	Y	P	G	A	A	**H**
VIM-13	E	A	E	G	N	E	V	P	T	H	S	L	E	G	L	S	S	S	G	D	A	V	R	F	G	P	V	E	L	F	Y	P	G	A	A	**H**
VIM-14	E	A	E	G	N	E	I	P	T	H	S	L	E	G	L	S	S	S	G	D	A	V	R	F	G	P	V	E	L	F	Y	P	G	A	A	**H**
VIM-15	E	V	E	G	N	E	I	P	T	H	S	L	E	G	L	S	S	S	G	D	A	V	R	F	G	P	V	E	L	F	Y	P	G	A	A	**H**
VIM-16	E	V	E	G	N	E	I	P	T	H	S	L	E	G	L	S	S	S	G	D	A	V	R	F	G	P	V	E	L	F	Y	P	G	A	A	**H**
VIM-17	E	V	E	G	N	E	I	P	T	H	S	L	E	G	L	S	S	S	G	D	A	V	R	F	G	P	V	E	L	F	Y	P	G	A	A	**H**
VIM-18	-	-	-	-	N	E	I	P	T	H	S	L	E	G	L	S	S	S	G	D	A	V	R	F	G	P	V	E	L	F	Y	P	G	A	A	**H**
VIM-19	E	A	E	G	N	E	I	P	T	H	S	L	E	G	L	S	S	S	G	D	A	V	R	F	G	P	V	E	L	F	Y	P	G	A	A	**H**
VIM-23	E	V	E	G	N	E	I	P	T	H	S	L	E	G	L	S	S	S	G	D	A	V	R	F	G	P	V	E	L	F	Y	P	G	A	A	**H**
VIM-24	E	V	E	G	N	E	I	P	T	H	S	L	E	G	L	S	S	S	G	D	A	V	R	F	G	P	V	E	L	F	Y	P	G	A	A	**H**
VIM-25	E	A	E	G	N	E	I	P	T	H	S	L	E	G	L	S	S	S	G	D	A	V	R	F	G	P	V	E	L	F	Y	P	G	A	A	**H**
VIM-26	E	A	E	G	N	E	I	P	T	H	S	L	E	G	L	S	S	S	G	D	A	V	R	F	G	P	V	E	L	F	Y	P	G	A	A	**H**
VIM-27	E	A	E	G	N	E	I	P	T	H	S	L	E	G	L	S	S	S	G	D	A	V	R	F	G	P	V	E	L	F	Y	P	G	A	A	**H**
VIM-28	E	A	E	G	N	E	I	P	T	H	S	L	E	G	L	S	S	S	G	D	A	V	R	F	G	P	V	E	L	F	Y	P	G	A	A	**H**
VIM-29	E	A	E	G	N	E	I	P	T	H	S	L	E	G	L	S	S	S	G	D	A	V	R	F	G	P	V	E	L	F	Y	P	G	A	A	**H**
VIM-30	E	V	E	G	N	E	I	P	T	H	S	L	E	G	L	S	S	S	G	D	A	V	R	F	G	P	V	E	L	F	Y	P	G	A	A	**H**
VIM-31	E	V	E	G	N	E	I	P	T	H	S	L	E	G	L	S	S	S	G	D	A	V	R	F	G	P	V	E	L	F	Y	P	G	A	A	**H**
VIM-32	E	A	A	G	N	E	I	P	T	H	S	L	E	G	L	S	S	S	G	D	A	V	R	F	G	P	V	E	L	F	Y	P	G	A	A	**H**
VIM-33	E	A	E	G	N	E	I	P	T	H	S	L	E	G	L	S	S	S	G	D	A	V	R	F	G	P	V	E	L	F	Y	P	G	A	A	**H**
VIM-34	E	A	E	G	N	E	I	P	T	H	S	L	E	G	L	S	S	S	G	D	A	V	R	F	G	P	V	E	L	F	Y	P	G	A	A	**H**
VIM-35	E	A	E	G	N	E	I	P	T	H	S	L	E	G	L	S	S	S	G	D	A	V	R	F	G	P	V	E	L	F	Y	P	G	A	A	**H**
VIM-36	E	V	E	G	N	E	I	P	T	H	S	L	E	G	L	S	S	S	G	D	A	V	R	F	G	P	V	E	L	F	Y	P	G	A	A	**H**
VIM-37	E	A	E	G	N	E	I	P	T	H	S	L	E	G	L	S	S	S	G	D	A	V	R	F	G	P	V	E	L	F	Y	P	G	A	A	**H**
VIM-38	E	A	E	G	N	E	I	P	T	H	S	L	E	G	L	S	S	S	G	D	A	V	R	F	G	P	V	E	L	F	Y	P	G	A	A	**H**

Gray: Signal peptide; Red: Zn(II) ligands; Green: positions related to substrate preferences

VIM variants protein sequence alignment- BBL numbering

	197	198	199	200	201	202	203	204	205	206	207	208	215	216	217	218	219	220	**221**	222	223	224	225	226	227	228	229	230	231	232	233	234	235	236	237	238
VIM-1	S	T	D	N	L	V	V	Y	V	P	S	A	N	V	L	Y	G	G	**C**	A	V	H	E	L	S	S	T	S	A	G	N	V	A	D	A	D
VIM-2	S	T	D	N	L	V	V	Y	V	P	S	A	S	V	L	Y	G	G	**C**	A	I	Y	E	L	S	R	T	S	A	G	N	V	A	D	A	D
VIM-3	S	T	D	N	L	V	V	Y	V	P	S	A	S	V	L	Y	G	G	**C**	A	I	Y	E	L	S	R	T	S	A	G	N	V	A	D	A	D
VIM-4	S	T	D	N	L	V	V	Y	V	P	S	A	N	V	L	Y	G	G	**C**	A	V	H	E	L	S	R	T	S	A	G	N	V	A	D	A	D
VIM-5	S	T	D	N	L	V	V	Y	V	P	S	A	N	V	L	Y	G	G	**C**	A	V	L	A	L	S	R	T	S	A	G	N	V	A	D	A	D
VIM-6	S	T	D	N	L	V	V	Y	V	P	S	A	S	V	L	Y	G	G	**C**	A	I	Y	E	L	S	R	T	S	A	G	N	V	A	D	A	D
VIM-7	S	G	D	N	L	V	V	Y	V	P	A	V	R	V	L	F	G	G	**C**	A	V	H	E	A	S	R	E	S	A	G	N	V	A	D	A	N
VIM-8	S	T	D	N	L	V	V	Y	V	P	S	A	S	V	L	Y	G	G	**C**	A	I	Y	E	L	S	R	T	S	A	G	N	V	A	D	A	D
VIM-9	S	T	D	N	L	V	V	Y	V	P	S	A	S	V	L	Y	G	G	**C**	A	I	Y	E	L	S	R	T	S	A	G	N	V	A	D	A	D
VIM-10	S	T	D	N	L	V	V	Y	V	P	S	A	S	V	L	Y	G	G	**C**	A	I	Y	E	L	S	R	T	S	A	G	N	V	A	D	A	D
VIM-11	S	T	D	N	L	V	V	Y	V	P	S	A	S	V	L	Y	G	G	**C**	A	I	Y	E	L	S	R	T	S	A	G	N	V	A	D	A	D
VIM-12	S	T	D	N	L	V	V	Y	V	P	S	A	N	V	L	Y	G	G	**C**	A	V	H	E	L	S	S	T	S	A	G	N	V	A	D	A	D
VIM-13	S	T	D	N	L	V	V	Y	V	P	S	A	N	V	L	Y	G	G	**C**	A	V	L	E	L	S	R	T	S	A	G	N	V	A	D	A	D
VIM-14	S	T	D	N	L	V	V	Y	V	P	S	A	N	V	L	Y	G	G	**C**	A	V	H	E	L	S	R	T	S	A	G	N	V	A	D	A	D
VIM-15	S	T	D	N	L	V	V	Y	V	P	S	A	S	V	L	F	G	G	**C**	A	I	Y	E	L	S	R	T	S	A	G	N	V	A	D	A	D
VIM-16	S	T	D	N	L	V	V	Y	V	P	S	A	S	V	L	Y	G	G	**C**	A	I	Y	E	L	S	R	T	S	A	G	N	V	A	D	A	D
VIM-17	S	T	D	N	L	V	V	Y	V	P	S	A	S	V	L	Y	G	G	**C**	A	I	Y	E	L	S	R	T	S	A	G	N	V	A	D	A	D
VIM-18	S	T	D	N	L	V	V	Y	V	P	S	A	S	V	L	Y	G	G	**C**	A	I	Y	E	L	S	R	T	S	A	G	N	V	A	D	A	D
VIM-19	S	T	D	N	L	V	V	Y	V	P	S	A	K	V	L	Y	G	G	**C**	A	V	H	E	L	S	R	T	S	A	G	N	V	A	D	A	D
VIM-23	S	T	D	N	L	V	V	Y	V	P	S	A	S	V	L	Y	G	G	**C**	A	I	Y	E	L	S	S	T	S	A	G	N	V	A	D	A	D
VIM-24	S	T	D	N	L	V	V	Y	V	P	S	A	S	V	L	Y	G	G	**C**	A	I	Y	E	L	S	L	T	S	A	G	N	V	A	D	A	D
VIM-25	S	T	D	N	L	V	V	Y	V	P	S	A	N	V	L	Y	G	G	**C**	A	V	L	A	L	S	R	T	S	A	G	N	V	A	D	A	D
VIM-26	S	T	D	N	L	V	V	Y	V	P	S	A	N	V	L	Y	G	G	**C**	A	V	L	E	L	S	S	T	S	A	G	N	V	A	D	A	D
VIM-27	S	T	D	N	L	V	V	Y	V	P	S	A	N	V	L	Y	G	G	**C**	A	V	H	E	L	S	S	T	S	A	G	N	V	A	D	A	D
VIM-28	S	T	D	N	L	V	V	Y	V	P	S	A	N	V	L	Y	G	G	**C**	A	V	L	E	L	S	R	T	S	A	G	N	V	A	D	A	D
VIM-29	S	T	D	N	L	V	V	Y	V	P	S	A	K	V	L	Y	G	G	**C**	A	V	H	E	L	S	S	T	S	A	G	N	V	A	D	A	D
VIM-30	S	T	D	N	L	V	V	Y	V	P	S	A	S	V	L	Y	G	G	**C**	A	I	Y	E	L	S	R	T	S	A	G	N	V	A	D	A	D
VIM-31	S	T	D	N	L	V	V	Y	V	P	S	A	S	V	L	Y	G	G	**C**	A	I	H	E	L	S	R	T	S	A	G	N	V	A	D	A	D
VIM-32	S	T	D	N	L	V	V	Y	V	P	S	A	N	V	L	Y	G	G	**C**	A	V	H	E	L	S	S	T	S	A	G	N	V	A	D	A	D
VIM-33	S	T	D	N	L	V	V	Y	V	P	S	A	N	V	L	F	G	G	**C**	A	V	H	E	L	S	S	T	S	A	G	N	V	A	D	A	D
VIM-34	S	T	D	N	L	V	V	Y	V	P	S	A	N	V	L	Y	G	G	**C**	A	V	H	E	L	S	S	T	S	A	G	N	V	A	D	A	D
VIM-35	S	T	D	N	L	V	V	Y	V	P	S	A	N	V	L	Y	G	G	**C**	A	V	H	E	L	S	S	T	S	A	G	N	V	T	D	A	D
VIM-36	S	T	D	N	L	V	V	Y	V	P	S	A	S	V	L	Y	G	G	**C**	A	I	Y	E	L	S	R	T	S	A	G	N	V	A	D	A	D
VIM-37	S	T	D	N	L	V	V	Y	V	P	S	A	N	V	L	Y	G	G	**C**	A	V	H	E	L	S	R	T	S	A	G	N	V	A	D	A	D
VIM-38	S	T	D	N	L	V	V	Y	V	P	S	A	N	V	L	Y	G	G	**C**	A	V	L	A	L	S	R	T	S	A	G	N	V	A	D	A	D

Gray: Signal peptide; Red: Zn(II) ligands; Green: positions related to substrate preferences

VIM variants protein sequence alignment- BBL numbering

	241	242	243	244	245	246	247	248	249	250	251	252	253	254	a	b	255	256	257	258	259	260	261	262	263	264	265	266	295	296	297	298	299	300	301	302
VIM-1	L	A	E	W	P	T	S	V	E	R	I	Q	K	H	Y	P	E	A	E	V	V	I	P	G	H	G	L	P	G	G	L	D	L	L	Q	H
VIM-2	L	A	E	W	P	T	S	I	E	R	I	Q	Q	H	Y	P	E	A	Q	F	V	I	P	G	H	G	L	P	G	G	L	D	L	L	K	H
VIM-3	L	A	E	W	P	T	S	I	E	R	I	Q	Q	H	Y	P	E	A	Q	F	V	I	P	G	H	G	L	P	G	G	L	D	L	L	K	H
VIM-4	L	A	E	W	P	T	S	V	E	R	I	Q	K	H	Y	P	E	A	E	V	V	I	P	G	H	G	L	P	G	G	L	D	L	L	Q	H
VIM-5	L	A	E	W	P	T	S	V	E	R	I	Q	K	H	Y	P	E	A	E	V	V	I	P	G	H	G	L	P	G	G	L	D	L	L	Q	H
VIM-6	L	A	E	W	P	T	S	I	E	R	I	Q	Q	H	Y	P	E	A	Q	F	V	I	P	G	H	G	L	P	G	G	L	D	L	L	K	H
VIM-7	L	A	E	W	P	A	T	I	K	R	I	Q	Q	R	Y	P	E	A	E	V	V	I	P	G	H	G	L	P	G	G	L	E	L	L	Q	H
VIM-8	L	A	E	W	P	T	S	I	E	R	I	Q	Q	H	Y	P	E	A	Q	F	V	I	P	G	H	G	L	P	G	G	L	D	L	L	K	H
VIM-9	L	A	E	W	P	T	S	I	E	R	I	Q	Q	H	Y	P	E	A	Q	F	V	I	P	G	H	G	L	P	G	G	L	D	L	L	K	H
VIM-10	L	A	E	W	P	T	S	I	E	R	I	Q	Q	H	Y	P	E	A	Q	Y	V	I	P	G	H	G	L	P	G	G	L	D	L	L	K	H
VIM-11	L	A	E	W	P	T	S	I	E	R	I	Q	Q	H	Y	P	E	A	Q	F	V	I	P	G	H	G	L	P	G	G	L	D	L	L	K	H
VIM-12	L	A	E	W	P	T	S	I	E	R	I	Q	Q	H	Y	P	E	A	Q	F	V	I	P	G	H	G	L	P	G	G	L	D	L	L	K	H
VIM-13	L	A	E	W	P	G	S	V	E	R	I	Q	Q	H	Y	P	E	A	E	V	V	I	P	G	H	G	L	P	G	G	L	D	L	L	Q	H
VIM-14	L	A	E	W	P	T	S	V	E	R	I	Q	K	H	Y	P	E	A	E	V	V	I	P	G	H	G	L	P	G	G	L	D	L	L	Q	H
VIM-15	L	A	E	W	P	T	S	I	E	R	I	Q	Q	H	Y	P	E	A	Q	F	V	I	P	G	H	G	L	P	G	G	L	D	L	L	K	H
VIM-16	L	A	E	W	P	T	S	I	E	R	I	Q	Q	H	Y	P	E	A	Q	F	V	I	P	G	H	G	L	P	G	G	L	D	L	L	K	H
VIM-17	L	A	E	W	P	T	S	I	E	R	I	Q	Q	H	Y	P	E	A	Q	F	V	I	P	G	H	G	L	P	G	G	L	D	L	L	K	H
VIM-18	L	A	E	W	P	T	S	I	E	R	I	Q	Q	H	Y	P	E	A	Q	F	V	I	P	G	H	G	L	P	G	G	L	D	L	L	K	H
VIM-19	L	A	E	W	P	T	S	V	E	R	I	Q	K	H	Y	P	E	A	E	V	V	I	P	G	H	G	L	P	G	G	L	D	L	L	Q	H
VIM-23	L	A	E	W	P	T	S	I	E	R	I	Q	Q	H	Y	P	E	A	Q	F	V	I	P	G	H	G	L	P	G	G	L	D	L	L	K	H
VIM-24	L	A	E	W	P	T	S	I	E	R	I	Q	Q	H	Y	P	E	A	Q	F	V	I	P	G	H	G	L	P	G	G	L	D	L	L	K	H
VIM-25	L	A	E	W	P	T	S	V	E	R	I	Q	K	H	Y	P	E	A	Q	F	V	I	P	G	H	G	L	P	G	G	L	D	L	L	K	H
VIM-26	L	A	E	W	P	T	S	V	E	R	I	Q	K	H	Y	P	E	A	E	V	V	I	P	G	H	G	L	P	G	G	L	D	L	L	Q	H
VIM-27	L	A	E	W	P	T	S	V	E	R	I	Q	K	H	Y	P	E	A	E	V	V	I	P	G	H	G	L	P	G	G	L	D	L	L	Q	H
VIM-28	L	A	E	W	P	T	S	V	E	R	I	Q	K	H	Y	P	E	A	E	V	V	I	P	G	H	G	L	P	G	G	L	D	L	L	Q	H
VIM-29	L	A	E	W	P	T	S	V	E	R	I	Q	K	R	Y	P	E	A	E	V	V	I	P	G	H	G	L	P	G	G	L	D	L	L	Q	H
VIM-30	L	A	E	W	P	T	S	I	E	R	I	Q	Q	H	Y	P	E	A	Q	F	V	I	P	G	H	G	L	P	G	G	L	D	L	L	K	H
VIM-31	L	A	E	W	P	T	S	I	E	R	I	Q	Q	R	Y	P	E	A	Q	F	V	I	P	G	H	G	L	P	G	G	L	D	L	L	K	H
VIM-32	L	A	E	W	P	T	S	V	E	R	I	Q	K	H	Y	P	E	A	E	V	V	I	P	G	H	G	L	P	G	G	L	D	L	L	Q	H
VIM-33	L	A	E	W	P	T	S	V	E	R	I	Q	K	H	Y	P	E	A	E	V	V	I	P	G	H	G	L	P	G	G	L	D	L	L	Q	H
VIM-34	L	A	E	W	P	T	S	V	E	R	I	Q	K	H	Y	P	E	A	E	V	V	I	P	G	H	G	L	P	G	G	L	D	L	L	Q	H
VIM-35	L	A	E	W	P	T	S	V	E	R	I	Q	K	H	Y	P	E	A	E	V	V	I	P	G	H	G	L	P	G	G	L	D	L	L	Q	H
VIM-36	L	A	E	W	P	T	S	I	E	R	I	Q	Q	H	Y	P	E	A	Q	F	V	I	P	G	H	G	L	P	G	G	L	D	L	L	K	H
VIM-37	L	A	E	W	P	T	S	V	E	R	I	Q	K	H	Y	P	E	A	E	V	V	I	P	G	H	G	L	P	G	G	L	D	L	L	Q	H
VIM-38	L	A	E	W	P	T	S	V	E	R	I	Q	K	H	Y	P	E	A	E	V	V	I	P	G	H	G	L	P	G	G	L	D	L	L	Q	H

Gray: Signal peptide; Red: Zn(II) ligands; Green: positions related to substrate preferences

VIM variants protein sequence alignment- BBL numbering

	303	304	305	306	307	308	309	310	311	312	313	314	315	316	317
VIM-1	T	A	N	V	V	K	A	H	K	N	R	S	V	A	E
VIM-2	T	T	N	V	V	K	A	H	T	N	R	S	V	V	E
VIM-3	T	T	N	V	V	K	A	H	T	N	R	S	V	V	E
VIM-4	T	A	N	V	V	K	A	H	K	N	R	S	V	A	E
VIM-5	T	A	N	V	V	T	A	H	K	N	R	S	V	A	E
VIM-6	T	T	N	V	V	K	A	H	T	N	R	S	V	V	E
VIM-7	T	T	N	V	V	K	T	H	K	V	R	P	V	A	E
VIM-8	T	T	N	V	V	K	A	H	T	N	R	S	V	V	E
VIM-9	T	T	N	V	V	K	A	H	T	N	R	S	V	V	E
VIM-10	T	T	N	V	V	K	A	H	T	N	R	S	V	V	E
VIM-11	T	T	N	V	V	K	A	H	T	N	R	S	V	V	E
VIM-12	T	T	N	V	V	K	A	H	T	N	R	S	V	V	E
VIM-13	T	A	N	V	V	K	A	H	T	N	R	S	V	A	E
VIM-14	T	A	N	V	V	K	A	H	K	N	R	S	V	A	E
VIM-15	T	T	N	V	V	K	A	H	T	N	R	S	V	V	E
VIM-16	T	T	N	V	V	K	A	H	T	N	R	S	V	V	E
VIM-17	T	T	N	V	V	K	A	H	T	N	R	S	V	V	E
VIM-18	T	T	N	V	V	K	A	H	T	N	R	S	V	V	E
VIM-19	T	A	N	V	V	K	A	H	K	N	R	S	V	A	E
VIM-23	T	T	N	V	V	K	A	H	T	N	R	S	V	V	E
VIM-24	T	T	N	V	V	K	A	H	T	N	R	S	V	V	E
VIM-25	T	T	N	V	V	K	A	H	T	N	R	S	V	V	E
VIM-26	T	A	N	V	V	K	A	H	K	N	R	S	V	A	E
VIM-27	T	A	N	V	V	K	A	H	K	N	R	S	V	A	E
VIM-28	T	A	N	V	V	K	A	H	K	N	R	S	V	A	E
VIM-29	T	A	N	V	V	K	A	H	K	N	R	S	V	A	E
VIM-30	T	T	N	V	V	K	A	H	T	N	R	S	V	V	E
VIM-31	T	T	N	V	V	K	A	H	T	N	R	S	V	V	E
VIM-32	T	A	N	V	V	K	A	H	K	N	R	S	V	A	E
VIM-33	T	A	N	V	V	K	A	H	K	N	R	S	V	A	E
VIM-34	T	A	N	V	V	K	A	H	K	N	R	S	V	A	E
VIM-35	T	A	N	V	V	K	A	H	K	N	R	S	V	A	E
VIM-36	T	T	N	V	V	K	A	H	T	N	R	S	V	V	E
VIM-37	T	A	N	V	V	K	A	H	K	N	R	S	V	A	E
VIM-38	T	A	N	V	V	T	A	H	K	N	R	S	V	V	E

Gray: Signal peptide; Red: Zn(II) ligands; Green: positions related to substrate preferences
