# Supplementary material for: Evolution of Metallo-β-lactamases: Trends Revealed by Natural Diversity and in vitro Evolution
Source: Antibiotics (Basel). 2014 Jul 1;3(3):285–316. doi: 10.3390/antibiotics3030285 (PMC4212336; doi:10.3390/antibiotics3030285)
Supplement: Supplementary File 2 [file antibiotics-03-00285-s002.pdf]

IMP variants protein sequence alignment- BBL numbering

	19	20	21	22	23	24	25	26	27	28	29	30	31	32	33	34	35	36	37	38	39	40	41	42	43	44	45	46	48	49	50	51	52	52	53	54
IMP-1	M	S	K	L	S	V	F	F	I	F	L	F	C	S	I	A	T	A	A	E	S	L	P	D	L	K	I	E	K	L	D	E	G	V	Y	V
IMP-2	M	K	K	L	F	V	L	C	V	C	F	L	C	S	I	T	A	A	G	A	R	L	P	D	L	K	I	E	K	L	E	E	G	V	Y	V
IMP-3	M	S	K	L	S	V	F	F	I	F	L	F	C	S	I	A	T	A	A	E	S	L	P	D	L	K	I	E	K	L	D	E	G	V	Y	V
IMP-4	M	S	K	L	S	V	F	F	I	F	L	F	C	S	I	A	T	A	A	E	P	L	P	D	L	K	I	E	K	L	D	E	G	V	Y	V
IMP-5	M	S	K	L	F	V	F	F	M	F	L	F	C	S	I	T	A	A	A	E	S	L	P	D	L	K	I	E	K	L	D	E	G	V	Y	V
IMP-6	M	S	K	L	S	V	F	F	I	F	L	F	C	S	I	A	T	A	A	E	S	L	P	D	L	K	I	E	K	L	D	E	G	V	Y	V
IMP-7	M	K	K	L	S	V	F	F	M	F	L	F	C	S	I	A	A	S	G	E	A	L	P	D	L	K	I	E	K	L	D	E	G	V	Y	V
IMP-8	M	K	K	L	F	V	L	C	V	C	F	L	C	S	I	T	A	A	G	A	A	L	P	D	L	K	I	E	K	L	E	E	G	V	Y	V
IMP-9	M	S	K	L	F	V	F	F	M	F	L	F	C	S	I	T	A	A	G	E	S	L	P	D	L	K	I	E	K	L	D	E	G	V	Y	V
IMP-10	M	S	K	L	S	V	F	F	I	F	L	F	C	S	I	A	T	A	A	E	S	L	P	D	L	K	I	E	K	L	D	E	G	V	Y	V
IMP-11	M	K	K	L	F	V	L	C	I	F	L	F	C	S	I	T	A	A	G	A	S	L	P	D	L	K	I	E	K	L	E	E	G	V	Y	V
IMP-12	M	K	K	L	F	V	L	C	I	F	L	F	L	S	I	T	A	S	G	E	V	L	P	D	L	K	I	E	K	L	E	E	G	V	Y	L
IMP-13	M	K	K	L	F	V	L	C	V	C	F	F	C	S	I	T	A	A	G	A	A	L	P	D	L	K	I	E	K	L	E	E	G	V	F	V
IMP-14	M	K	K	L	F	V	L	C	V	F	F	F	C	N	I	A	V	A	E	E	S	L	P	D	L	K	I	E	K	L	E	E	G	V	Y	V
IMP-15	M	N	K	L	S	V	F	F	M	F	M	F	C	S	I	T	A	A	G	E	S	L	P	D	L	K	I	E	K	L	D	E	G	V	Y	V
IMP-16	M	K	K	L	F	V	L	C	I	F	L	F	C	S	I	T	A	A	G	E	S	L	P	D	L	K	I	E	K	L	E	D	G	V	Y	V
IMP-18	M	K	K	L	F	V	L	C	V	F	F	L	C	N	I	A	A	A	D	D	S	L	P	D	L	K	I	E	K	L	E	K	G	V	Y	V
IMP-19	M	K	K	L	F	V	L	C	V	C	F	L	C	S	I	T	A	A	G	A	A	L	P	D	L	K	I	E	K	L	E	E	G	V	Y	V
IMP-20	M	K	K	L	F	V	L	C	V	C	F	L	C	S	I	T	A	A	G	A	A	L	P	D	L	K	I	E	K	L	E	E	G	V	Y	V
IMP-21	M	K	K	L	F	V	L	C	I	F	L	F	C	S	I	T	A	A	G	A	S	L	P	D	L	K	I	E	K	L	E	E	G	V	Y	V
IMP-22	M	K	K	L	F	V	L	C	V	F	L	F	C	S	I	T	A	A	G	E	S	L	P	D	L	K	I	E	K	L	E	E	G	V	Y	V
IMP-24	M	K	K	L	F	V	L	C	V	C	F	L	C	S	I	T	A	A	G	A	A	L	P	D	L	K	I	E	K	L	E	E	G	V	Y	V
IMP-25	M	S	K	L	S	V	F	F	I	F	L	F	C	S	I	A	T	A	A	E	S	L	P	D	L	K	I	E	K	L	D	E	G	V	Y	V
IMP-26	M	S	K	L	S	V	F	F	I	F	L	F	C	S	I	A	T	A	A	E	P	L	P	D	L	K	I	E	K	L	D	E	G	V	Y	V
IMP-27	M	K	K	L	F	V	L	C	V	F	V	F	C	S	I	T	V	A	G	E	T	L	P	N	L	R	V	E	K	L	E	E	G	V	Y	V
IMP-28	M	S	K	L	F	V	F	F	M	F	L	F	C	S	I	T	A	A	A	E	S	L	P	D	L	K	I	E	R	L	D	E	G	V	Y	V
IMP-29	M	S	K	L	F	V	F	L	I	F	L	F	C	S	I	T	A	A	A	E	S	L	P	D	L	K	I	E	K	L	D	E	G	V	Y	V
IMP-30	M	S	K	L	S	V	F	F	I	F	L	F	C	S	I	A	T	A	A	E	S	L	P	D	L	K	I	E	K	L	D	E	G	V	Y	V
IMP-31	M	K	K	I	F	V	L	F	V	F	L	F	C	S	I	T	A	A	G	E	S	L	P	D	I	K	I	E	K	L	D	E	D	V	Y	V
IMP-32	M	K	K	L	F	V	L	C	V	F	F	F	C	N	I	A	V	A	E	E	S	L	P	D	L	K	I	E	K	L	E	E	G	V	Y	V
IMP-33	M	K	K	L	F	V	L	C	V	C	F	F	C	S	I	T	A	A	G	S	S	L	P	D	L	K	I	E	K	L	E	E	G	V	F	V
IMP-34	M	S	K	L	S	V	F	F	I	F	L	F	C	S	I	A	T	A	A	E	S	L	P	D	L	K	I	E	K	L	D	E	G	V	Y	V
IMP-35	M	K	K	I	F	V	L	F	V	F	L	F	C	S	I	T	A	A	G	E	S	L	P	D	I	K	I	E	K	L	D	E	D	V	Y	V
IMP-37	M	K	K	L	F	V	L	C	V	C	F	F	C	S	I	T	A	A	G	A	A	L	P	D	L	K	I	E	K	L	E	E	G	V	F	V
IMP-38	M	S	K	L	S	V	F	F	I	F	L	F	C	S	I	A	T	A	A	E	P	L	P	D	L	K	I	E	K	L	D	E	G	V	Y	V
IMP-40	M	S	K	L	S	V	F	F	I	F	L	F	C	S	I	A	T	A	A	E	S	L	P	D	L	K	I	E	K	L	D	E	G	V	Y	V
IMP-41	M	K	K	L	F	V	L	C	I	F	L	F	C	S	I	T	A	A	G	A	S	L	P	D	L	K	I	E	K	L	E	E	G	V	Y	V
IMP-42	M	S	K	L	S	V	F	F	I	F	L	F	C	S	I	A	T	A	A	E	S	L	P	D	L	K	I	E	K	L	D	E	G	V	Y	V
IMP-43	M	K	K	L	S	V	F	F	M	F	L	F	C	S	I	A	A	S	G	E	A	L	P	D	L	K	I	E	K	L	D	E	G	V	Y	V
IMP-44	M	K	K	L	F	V	L	C	I	F	L	F	C	S	I	T	A	A	G	A	S	L	P	D	L	K	I	E	K	L	E	E	G	V	Y	V

Gray: Signal peptide; Red: Zn(II) ligands; Green: positions related to substrate preferences; Ligh Blue: positions that tolerate variations with minnor susbtrate dependance

IMP variants protein sequence alignment- BBL numbering

	55	56	57	58	59	60	61	62	63	64	65	66	67	68	69	70	71	72	73	74	75	76	77	78	79	80	81	82	83	84	85	86	87	88	89	90
IMP-1	H	T	S	F	E	E	V	N	G	W	G	V	V	P	K	H	G	L	V	V	L	V	N	A	E	A	Y	L	I	D	T	P	F	T	A	K
IMP-2	H	T	S	F	E	E	V	N	G	W	G	V	V	S	K	H	G	L	V	V	L	V	N	T	D	A	Y	L	I	D	T	P	F	T	A	T
IMP-3	H	T	S	F	E	E	V	N	G	W	G	V	V	P	K	H	G	L	V	V	L	V	N	A	E	A	Y	L	I	D	T	P	F	T	A	K
IMP-4	H	T	S	F	E	E	V	N	G	W	G	V	V	P	K	H	G	L	V	V	L	V	D	A	E	A	Y	L	I	D	T	P	F	T	A	K
IMP-5	H	T	S	F	E	E	V	N	G	W	G	V	V	P	K	H	G	L	V	V	L	V	N	T	E	A	Y	L	I	D	T	P	F	T	A	K
IMP-6	H	T	S	F	E	E	V	N	G	W	G	V	V	P	K	H	G	L	V	V	L	V	N	A	E	A	Y	L	I	D	T	P	F	T	A	K
IMP-7	H	T	S	F	E	E	V	N	G	W	G	V	V	P	K	H	G	L	V	V	L	V	N	T	D	A	Y	L	I	D	T	P	F	T	A	K
IMP-8	H	T	S	F	E	E	V	N	G	W	G	V	V	S	K	H	G	L	V	V	L	V	N	T	D	A	Y	L	I	D	T	P	F	T	A	T
IMP-9	H	T	S	F	E	E	V	N	G	W	G	V	I	P	K	H	G	L	V	V	L	V	N	T	D	A	Y	L	I	D	T	P	F	T	A	K
IMP-10	H	T	S	F	E	E	V	N	G	W	G	V	F	P	K	H	G	L	V	V	L	V	N	A	E	A	Y	L	I	D	T	P	F	T	A	K
IMP-11	H	T	S	F	E	E	V	N	G	W	G	V	V	S	K	H	G	L	V	V	L	V	N	T	D	A	Y	L	I	D	T	P	F	T	A	K
IMP-12	H	T	S	F	E	E	V	S	G	W	G	V	V	T	K	H	G	L	V	V	L	V	N	N	D	A	Y	L	I	D	T	P	F	T	N	K
IMP-13	H	T	S	F	E	E	V	N	G	W	G	V	V	T	K	H	G	L	V	V	L	V	N	T	D	A	Y	L	I	D	T	P	F	T	A	T
IMP-14	H	T	S	F	E	E	V	K	G	W	S	V	V	T	K	H	G	L	V	V	L	V	K	N	D	A	Y	L	I	D	T	P	I	T	A	K
IMP-15	H	T	S	F	E	E	V	N	G	W	G	V	V	P	K	H	G	L	V	V	L	V	N	T	E	A	Y	L	I	D	T	P	F	T	A	K
IMP-16	H	T	S	F	E	E	V	N	G	W	G	V	V	T	K	H	G	L	V	F	L	V	N	T	D	A	Y	L	I	D	T	P	F	A	A	K
IMP-18	H	T	S	F	E	E	V	K	G	W	G	V	V	T	K	H	G	L	V	V	L	V	K	N	D	A	Y	L	I	D	T	P	I	T	A	K
IMP-19	H	T	S	F	E	E	V	N	G	W	G	V	V	S	K	H	G	L	V	V	L	V	N	T	D	A	Y	L	I	D	T	P	F	T	A	T
IMP-20	H	T	S	F	E	E	V	N	G	W	G	V	F	S	K	H	G	L	V	V	L	V	N	T	D	A	Y	L	I	D	T	P	F	T	A	T
IMP-21	H	T	S	F	E	E	V	N	G	W	G	V	A	S	K	H	G	L	V	V	L	V	N	T	D	A	Y	L	I	D	T	P	F	T	A	K
IMP-22	H	T	S	F	E	E	V	N	G	W	G	V	V	S	K	H	G	L	V	I	L	V	N	T	D	A	Y	L	I	D	T	P	F	T	A	K
IMP-24	H	T	S	F	E	E	V	N	G	W	G	V	V	S	K	H	G	L	V	V	L	V	N	T	D	A	Y	L	I	D	T	P	F	T	A	T
IMP-25	H	T	S	F	E	E	V	N	G	W	G	V	V	P	K	H	G	L	V	V	L	V	N	A	E	A	Y	L	I	D	T	P	F	T	A	K
IMP-26	H	T	S	F	E	E	V	N	G	W	G	V	F	P	K	H	G	L	V	V	L	V	D	A	E	A	Y	L	I	D	T	P	F	T	A	K
IMP-27	H	T	S	Y	E	E	V	K	G	W	G	V	V	T	K	H	G	L	V	V	L	I	G	A	D	A	Y	L	I	D	T	P	F	T	A	K
IMP-28	H	T	S	F	E	E	V	N	G	W	G	V	V	P	K	H	G	L	V	V	L	V	N	T	E	A	Y	L	I	D	T	P	F	T	A	K
IMP-29	H	T	S	F	E	E	V	N	G	W	G	V	V	P	K	H	G	L	V	V	L	V	N	T	E	A	Y	L	I	D	T	P	F	T	A	K
IMP-30	H	T	S	F	K	E	V	N	G	W	G	V	V	P	K	H	G	L	V	V	L	V	N	A	E	A	Y	L	I	D	T	P	F	T	A	K
IMP-31	H	T	S	F	E	K	I	T	G	W	G	V	I	T	K	H	G	L	V	V	L	V	N	T	D	A	Y	I	I	D	T	P	F	T	A	K
IMP-32	H	T	S	F	E	E	V	K	G	W	S	V	V	T	K	H	G	L	V	V	L	V	K	N	D	A	Y	L	I	D	T	P	I	T	A	K
IMP-33	H	T	S	F	E	E	V	N	G	W	G	V	V	T	K	H	G	L	V	V	L	V	N	T	D	A	Y	L	I	D	T	P	F	T	A	T
IMP-34	H	T	S	F	E	E	V	N	G	W	G	V	V	P	K	H	G	L	V	V	L	V	N	A	E	A	Y	L	I	D	T	P	F	T	A	K
IMP-35	H	T	S	F	E	E	D	N	G	W	G	V	I	T	K	H	G	L	V	V	L	V	N	T	D	A	Y	I	I	D	T	P	F	T	A	K
IMP-37	H	T	S	F	E	E	V	N	G	W	G	V	V	T	K	H	G	L	V	V	L	V	N	T	D	A	Y	L	I	D	T	P	F	T	A	T
IMP-38	H	T	S	F	E	E	V	N	G	W	G	V	V	P	K	H	G	L	V	V	L	V	D	A	E	A	Y	L	I	D	T	P	F	T	A	K
IMP-40	H	T	S	F	E	E	V	N	G	W	G	V	F	P	K	H	G	L	V	V	L	V	N	A	E	A	Y	L	I	D	T	P	S	T	A	K
IMP-41	H	T	S	F	E	E	V	N	G	W	G	V	F	S	K	H	G	L	V	V	L	V	N	T	D	A	Y	L	I	D	T	P	F	T	A	K
IMP-42	H	T	S	F	E	E	V	N	R	W	G	V	V	P	K	H	G	L	V	V	L	V	N	A	E	A	Y	L	I	D	T	P	F	T	A	K
IMP-43	H	T	S	F	E	E	V	N	G	W	G	V	F	P	K	H	G	L	V	V	L	V	N	T	D	A	Y	L	I	D	T	P	F	T	A	K
IMP-44	H	T	S	F	E	E	V	N	G	W	G	V	F	S	K	H	G	L	V	V	L	V	N	T	D	A	Y	L	I	D	T	P	S	T	A	K

Gray: Signal peptide; Red: Zn(II) ligands; Green: positions related to substrate preferences; Ligh Blue: positions that tolerate variations with minnor susbtrate dependance

IMP variants protein sequence alignment- BBL numbering

	91	92	93	94	95	96	97	98	99	100	101	102	103	104	108	109	110	111	112	113	114	115	**116**	117	**118**	119	**120**	121	122	123	124	125	126	127	128	129
IMP-1	D	T	E	K	L	V	T	W	F	V	E	R	G	Y	K	I	K	G	S	I	S	S	**H**	F	**H**	S	**D**	S	T	G	G	I	E	W	L	N
IMP-2	D	T	E	K	L	V	N	W	F	V	E	R	G	Y	K	I	K	G	T	I	S	S	**H**	F	**H**	S	**D**	S	T	G	G	I	E	W	L	N
IMP-3	D	T	E	K	L	V	T	W	F	V	E	R	G	Y	K	I	K	G	S	I	S	S	**H**	F	**H**	S	**D**	S	T	G	G	I	G	W	L	N
IMP-4	D	T	E	K	L	V	T	W	F	V	E	R	G	Y	K	I	K	G	S	I	S	S	**H**	F	**H**	S	**D**	S	T	G	G	I	E	W	L	N
IMP-5	D	T	E	K	L	V	T	W	F	V	E	R	G	Y	K	I	K	G	S	I	S	S	**H**	F	**H**	S	**D**	S	T	G	G	I	E	W	L	N
IMP-6	D	T	E	K	L	V	T	W	F	V	E	R	G	Y	K	I	K	G	S	I	S	S	**H**	F	**H**	S	**D**	S	T	G	G	I	E	W	L	N
IMP-7	D	T	E	K	L	V	T	W	F	V	E	R	G	Y	K	I	K	G	S	I	S	S	**H**	F	**H**	S	**D**	S	T	G	G	I	E	W	L	N
IMP-8	D	T	E	K	L	V	N	W	F	V	E	R	G	Y	K	I	K	G	T	I	S	S	**H**	F	**H**	S	**D**	S	T	G	G	I	E	W	L	N
IMP-9	D	T	E	N	L	V	N	W	F	V	E	R	G	Y	R	I	K	G	S	I	S	S	**H**	F	**H**	S	**D**	S	T	G	G	I	E	W	L	N
IMP-10	D	T	E	K	L	V	T	W	F	V	E	R	G	Y	K	I	K	G	S	I	S	S	**H**	F	**H**	S	**D**	S	T	G	G	I	E	W	L	N
IMP-11	D	T	E	K	L	V	N	W	F	V	E	R	G	Y	K	I	K	G	S	I	S	S	**H**	F	**H**	S	**D**	S	T	G	G	I	E	W	L	N
IMP-12	D	T	E	K	L	V	A	W	F	V	G	R	G	F	T	I	K	G	S	V	S	S	**H**	F	**H**	S	**D**	S	T	G	G	I	E	W	L	N
IMP-13	D	T	E	K	L	V	N	W	F	V	E	R	G	Y	E	I	K	G	T	I	S	S	**H**	F	**H**	S	**D**	S	T	G	G	I	E	W	L	N
IMP-14	D	T	E	K	L	V	N	W	F	V	E	R	G	Y	K	I	K	G	S	I	S	T	**H**	F	**H**	G	**D**	S	T	A	G	I	E	W	L	N
IMP-15	D	T	E	K	L	V	T	W	F	V	E	R	G	Y	K	I	K	G	S	I	S	S	**H**	F	**H**	S	**D**	S	T	G	G	I	E	W	L	N
IMP-16	D	T	E	K	L	V	N	W	F	V	E	R	G	Y	K	I	K	G	S	I	S	S	**H**	F	**H**	S	**D**	S	S	G	G	I	E	W	L	N
IMP-18	D	T	E	K	L	V	N	W	F	I	E	H	G	Y	R	I	K	G	S	I	S	T	**H**	F	**H**	G	**D**	S	T	A	G	I	E	W	L	N
IMP-19	D	T	E	K	L	V	N	W	F	V	E	R	G	Y	K	I	K	G	T	I	S	S	**H**	F	**H**	S	**D**	S	T	G	G	I	E	W	L	N
IMP-20	D	T	E	K	L	V	N	W	F	V	E	R	G	Y	K	I	K	G	T	I	S	S	**H**	F	**H**	S	**D**	S	T	G	G	I	E	W	L	N
IMP-21	D	T	E	K	L	V	N	W	F	V	E	R	G	Y	K	I	K	G	S	I	S	S	**H**	F	**H**	S	**D**	S	T	G	G	I	E	W	L	N
IMP-22	D	T	E	K	L	V	T	W	F	V	E	R	G	Y	K	I	K	G	S	I	S	S	**H**	F	**H**	S	**D**	S	T	G	G	I	E	W	L	N
IMP-24	D	T	E	K	L	V	N	W	F	V	E	R	G	Y	K	I	K	G	T	I	S	S	**H**	F	**H**	S	**D**	S	T	G	G	I	E	W	L	N
IMP-25	D	T	E	K	L	V	T	W	F	V	E	R	G	Y	K	I	K	G	S	I	S	S	**H**	F	**H**	S	**D**	S	T	G	G	I	E	W	L	N
IMP-26	D	T	E	K	L	V	T	W	F	V	E	R	G	Y	K	I	K	G	S	I	S	S	**H**	F	**H**	S	**D**	S	T	G	G	I	E	W	L	N
IMP-27	D	T	E	K	L	V	N	W	F	V	E	R	G	Y	K	I	K	G	T	V	S	S	**H**	F	**H**	S	**D**	S	T	G	G	I	E	W	L	N
IMP-28	D	T	E	K	L	V	T	W	F	V	G	R	G	Y	K	I	K	G	S	I	S	S	**H**	F	**H**	S	**D**	S	T	G	G	I	E	W	L	N
IMP-29	D	T	E	K	L	V	T	W	F	V	E	R	G	Y	K	I	K	G	S	I	S	S	**H**	F	**H**	S	**D**	S	T	G	G	I	E	W	L	N
IMP-30	D	T	E	K	L	V	T	W	F	V	E	R	G	Y	K	I	K	G	S	I	S	S	**H**	F	**H**	S	**D**	S	T	G	G	I	E	W	L	N
IMP-31	D	T	E	K	L	V	R	W	F	V	G	R	G	Y	K	I	K	G	S	I	S	S	**H**	F	**H**	S	**D**	S	A	G	G	I	E	W	L	N
IMP-32	D	T	E	K	L	V	N	W	F	V	E	R	G	Y	K	I	K	G	S	I	S	T	**H**	F	**H**	G	**D**	S	T	A	G	I	E	W	L	N
IMP-33	D	T	E	K	L	V	N	W	F	V	E	R	G	Y	K	I	K	G	T	I	S	S	**H**	F	**H**	S	**D**	S	T	G	G	I	E	W	L	N
IMP-34	D	T	E	K	L	V	T	W	F	V	E	R	G	Y	K	I	K	G	S	I	S	S	**H**	F	**H**	S	**D**	S	T	G	G	I	G	W	L	N
IMP-35	D	T	E	K	L	V	R	W	F	V	G	R	G	Y	K	I	K	G	S	I	S	S	**H**	F	**H**	S	**D**	S	A	G	G	I	E	W	L	N
IMP-37	D	T	E	K	L	V	N	W	F	V	E	R	G	Y	E	I	K	G	T	I	S	S	**H**	F	**H**	S	**D**	S	T	G	G	I	E	W	L	N
IMP-38	D	T	E	K	L	V	T	W	F	V	E	R	G	Y	K	I	K	G	S	I	S	S	**H**	F	**H**	S	**D**	S	T	G	G	I	E	W	L	N
IMP-40	D	T	E	K	L	V	T	W	F	V	E	R	G	Y	K	I	K	G	S	I	S	S	**H**	F	**H**	S	**D**	S	T	G	G	I	E	W	L	N
IMP-41	D	T	E	K	L	V	N	W	F	V	E	R	G	Y	K	I	K	G	S	I	S	S	**H**	F	**H**	S	**D**	S	T	G	G	I	E	W	L	N
IMP-42	D	T	E	K	L	V	T	W	F	V	E	R	G	Y	K	I	K	G	S	I	S	S	**H**	F	**H**	S	**D**	S	T	G	G	I	E	W	L	N
IMP-43	D	T	E	K	L	V	T	W	F	V	E	R	G	Y	K	I	K	G	S	I	S	S	**H**	F	**H**	S	**D**	S	T	G	G	I	E	W	L	N
IMP-44	D	T	E	K	L	V	N	W	F	V	E	R	G	Y	K	I	K	G	S	I	S	S	**H**	F	**H**	S	**D**	S	T	G	G	I	E	W	L	N

Gray: Signal peptide; Red: Zn(II) ligands; Green: positions related to substrate preferences; Ligh Blue: positions that tolerate variations with minnor susbtrate dependance

IMP variants protein sequence alignment- BBL numbering

	130	132	133	134	135	136	137	138	139	140	141	142	143	144	145	146	147	148	149	150	150a	167	168	169	170	171	172	173	174	175	176	177	178	179	180	181
IMP-1	S	R	S	I	P	T	Y	A	S	E	L	T	N	E	L	L	K	K	D	G	K	V	Q	A	T	N	S	F	S	G	V	N	Y	W	L	V
IMP-2	S	Q	S	I	P	T	Y	A	S	E	L	T	N	E	L	L	K	K	D	G	K	V	Q	A	K	N	S	F	S	G	V	S	Y	W	L	V
IMP-3	S	R	S	I	P	T	Y	A	S	E	L	T	N	E	L	L	K	K	D	G	K	V	Q	A	T	N	S	F	S	G	V	N	Y	W	L	V
IMP-4	S	Q	S	I	P	T	Y	A	S	E	L	T	N	E	L	L	K	K	D	G	K	V	Q	A	K	N	S	F	G	G	V	N	Y	W	L	V
IMP-5	S	Q	S	I	P	T	Y	A	S	E	L	T	N	E	L	L	K	K	D	G	K	V	Q	A	K	N	S	F	S	G	A	S	Y	W	L	V
IMP-6	S	R	S	I	P	T	Y	A	S	E	L	T	N	E	L	L	K	K	D	G	K	V	Q	A	T	N	S	F	S	G	V	N	Y	W	L	V
IMP-7	S	Q	S	I	P	T	Y	A	S	E	L	T	N	E	L	L	K	K	D	G	K	V	Q	A	K	N	S	F	S	G	A	S	Y	W	L	V
IMP-8	S	Q	S	I	P	T	Y	A	S	E	L	T	N	E	L	L	K	K	D	G	K	V	Q	A	K	N	S	F	S	G	V	S	Y	W	L	V
IMP-9	S	Q	S	I	P	T	Y	A	S	E	L	T	N	E	L	L	K	K	D	G	K	V	Q	A	K	Y	S	F	S	G	V	S	Y	W	L	V
IMP-10	S	R	S	I	P	T	Y	A	S	E	L	T	N	E	L	L	K	K	D	G	K	V	Q	A	T	N	S	F	S	G	V	N	Y	W	L	V
IMP-11	S	Q	S	I	P	T	Y	A	S	V	L	T	N	E	L	L	K	K	D	G	K	V	Q	A	K	N	S	F	S	G	V	S	Y	W	L	V
IMP-12	S	Q	S	I	P	T	Y	A	S	E	L	T	N	E	L	L	K	K	N	G	K	V	Q	A	T	N	S	F	S	G	V	S	Y	W	L	V
IMP-13	S	Q	S	I	P	T	Y	A	S	E	L	T	N	E	L	L	K	K	S	G	K	V	Q	A	K	Y	S	F	S	E	V	S	Y	W	L	V
IMP-14	S	Q	S	I	P	T	Y	A	S	E	L	T	N	E	L	L	K	K	D	N	K	V	Q	A	K	H	S	F	N	G	V	S	Y	S	L	I
IMP-15	S	Q	S	I	P	T	Y	A	S	E	L	T	N	E	L	L	K	K	D	G	K	V	Q	A	K	N	S	F	S	G	G	S	Y	W	L	V
IMP-16	S	Q	S	I	P	T	Y	A	S	E	L	T	N	E	L	L	K	K	N	G	K	V	Q	A	K	N	S	F	S	G	V	S	Y	W	L	L
IMP-18	S	Q	S	I	S	T	Y	A	S	E	L	T	N	E	L	L	K	K	D	N	K	V	Q	A	T	N	S	F	S	G	V	S	Y	S	L	I
IMP-19	S	Q	S	I	P	T	Y	A	S	E	L	T	N	E	L	L	K	K	D	G	K	V	Q	A	K	N	S	F	S	G	V	S	Y	W	L	V
IMP-20	S	Q	S	I	P	T	Y	A	S	E	L	T	N	E	L	L	K	K	D	G	K	V	Q	A	K	N	S	F	S	G	V	S	Y	W	L	V
IMP-21	S	Q	S	I	P	T	Y	A	S	V	L	T	N	E	L	L	K	K	D	G	K	V	Q	A	K	N	S	F	S	G	V	S	Y	W	L	V
IMP-22	S	Q	S	I	P	T	Y	A	S	E	L	T	N	D	L	L	K	Q	N	G	K	V	Q	A	K	N	S	F	S	G	V	S	Y	W	L	V
IMP-24	S	Q	S	I	P	T	Y	A	S	E	L	T	N	E	L	L	K	K	D	G	K	V	Q	A	K	N	S	F	S	G	V	S	Y	W	L	V
IMP-25	S	R	S	I	P	T	Y	A	S	E	L	T	N	E	L	L	K	K	D	G	K	V	Q	A	T	N	S	F	S	G	V	N	Y	W	L	V
IMP-26	S	Q	S	I	P	T	Y	A	S	E	L	T	N	E	L	L	K	K	D	G	K	V	Q	A	K	N	S	F	G	G	V	N	Y	W	L	V
IMP-27	S	Q	S	I	P	T	Y	A	S	E	L	T	N	E	L	L	K	K	D	G	K	V	Q	A	K	N	S	F	D	G	V	S	Y	W	L	A
IMP-28	S	Q	S	I	P	T	Y	A	S	E	L	T	N	E	L	L	K	K	D	G	K	V	Q	A	K	N	S	F	G	G	V	S	Y	W	L	V
IMP-29	S	Q	S	I	P	T	Y	A	S	E	L	T	N	E	L	L	K	K	G	G	K	V	Q	A	K	N	S	F	S	G	V	S	Y	W	L	V
IMP-30	S	R	S	I	P	T	Y	A	S	E	L	T	N	E	L	L	K	K	D	G	K	V	Q	A	T	N	S	F	S	G	V	N	Y	W	L	V
IMP-31	S	Q	S	I	P	T	Y	A	S	K	L	T	N	E	L	L	K	K	N	G	N	A	Q	A	E	N	S	F	S	G	V	S	Y	W	L	V
IMP-32	S	Q	S	I	P	T	Y	A	S	E	L	T	N	E	L	L	K	K	D	N	K	V	Q	A	K	H	S	F	Y	G	V	S	Y	S	L	I
IMP-33	S	Q	S	I	P	T	Y	A	S	E	L	T	N	E	L	L	K	K	S	G	K	V	Q	A	K	Y	S	F	S	E	V	S	Y	W	L	V
IMP-34	S	R	S	I	P	T	Y	A	S	E	L	T	N	E	L	L	K	K	D	G	K	V	Q	A	T	N	S	F	S	G	V	N	Y	W	L	V
IMP-35	S	Q	S	I	P	T	Y	A	S	K	L	T	N	E	L	L	K	K	N	G	N	A	Q	A	E	N	S	F	S	G	V	S	Y	W	L	V
IMP-37	S	Q	S	I	P	T	Y	A	S	E	L	T	N	E	L	L	K	K	S	G	K	V	Q	A	K	Y	S	F	S	E	V	S	Y	W	L	V
IMP-38	S	Q	S	I	P	T	Y	A	S	E	L	T	N	E	L	L	K	K	D	G	K	V	Q	A	K	N	S	F	G	G	V	N	Y	W	L	V
IMP-40	S	R	S	I	P	T	Y	A	S	E	L	T	N	E	L	L	K	K	D	G	K	V	Q	A	T	N	S	F	S	G	V	N	Y	W	L	V
IMP-41	S	Q	S	I	P	T	Y	A	S	V	L	T	N	E	L	L	K	K	D	G	K	V	Q	A	K	N	S	F	S	G	V	S	Y	W	L	V
IMP-42	S	R	S	I	P	T	Y	A	S	E	L	T	N	E	L	L	K	K	D	G	K	V	Q	A	T	N	S	F	S	G	V	N	Y	W	L	V
IMP-43	S	Q	S	I	P	T	Y	A	S	E	L	T	N	E	L	L	K	K	D	G	K	V	Q	A	K	N	S	F	S	G	A	S	Y	W	L	V
IMP-44	S	Q	S	I	P	T	Y	A	S	V	L	T	N	E	L	L	K	K	D	G	K	V	Q	A	K	N	S	F	S	G	V	S	Y	W	L	V

Gray: Signal peptide; Red: Zn(II) ligands; Green: positions related to substrate preferences; Ligh Blue: positions that tolerate variations with minnor susbtrate dependance

IMP variants protein sequence alignment- BBL numbering

	182	183	184	185	186	187	190	191	192	193	194	195	196	197	198	199	200	201	202	203	204	205	206	207	208	215	216	217	218	219	220	221	222	223	224	225
IMP-1	K	N	K	I	E	V	F	Y	P	G	P	G	H	T	P	D	N	V	V	V	W	L	P	E	R	K	I	L	F	G	G	C	F	I	K	P
IMP-2	K	N	K	I	E	V	F	Y	P	G	P	G	H	T	Q	D	N	V	V	V	W	L	P	E	K	K	I	L	F	G	G	C	F	V	K	P
IMP-3	K	N	K	I	E	V	F	Y	P	G	P	G	H	T	P	D	N	V	V	V	W	L	P	E	R	K	I	L	F	G	G	C	F	I	K	P
IMP-4	K	N	K	I	E	V	F	Y	P	G	P	G	H	T	P	D	N	L	V	V	W	L	P	E	R	K	I	L	F	G	G	C	F	I	K	P
IMP-5	K	K	K	I	E	V	F	Y	P	G	P	G	H	T	P	D	N	V	V	V	W	L	P	E	N	R	V	L	F	G	G	C	F	V	K	P
IMP-6	K	N	K	I	E	V	F	Y	P	G	P	G	H	T	P	D	N	V	V	V	W	L	P	E	R	K	I	L	F	G	G	C	F	I	K	P
IMP-7	K	K	K	I	E	I	F	Y	P	G	P	G	H	T	P	D	N	V	V	V	W	L	P	E	H	R	V	L	F	G	G	C	F	V	K	P
IMP-8	K	N	K	I	E	V	F	Y	P	G	P	G	H	T	Q	D	N	V	V	V	W	L	P	E	K	K	I	L	F	G	G	C	F	V	K	P
IMP-9	K	K	K	I	E	V	F	Y	P	G	P	G	H	A	P	D	N	V	V	V	W	L	P	E	N	R	V	L	F	G	G	C	F	V	K	P
IMP-10	K	N	K	I	E	V	F	Y	P	G	P	G	H	T	P	D	N	V	V	V	W	L	P	E	R	K	I	L	F	G	G	C	F	I	K	P
IMP-11	K	N	K	I	E	V	F	Y	P	G	P	G	H	T	Q	D	N	V	V	V	W	L	P	K	N	K	I	L	F	G	G	C	F	V	K	P
IMP-12	K	N	K	I	E	I	F	Y	P	G	P	G	H	T	Q	D	N	V	V	V	W	L	P	E	N	K	I	L	F	G	G	C	F	V	K	P
IMP-13	K	N	K	I	E	V	F	Y	P	G	P	G	H	T	Q	D	N	L	V	V	W	L	P	E	S	K	I	L	F	G	G	C	F	I	K	P
IMP-14	K	N	K	I	E	V	F	Y	P	G	P	G	H	T	Q	D	N	V	V	V	W	L	P	E	K	K	I	L	F	G	G	C	F	V	K	P
IMP-15	N	N	K	I	E	V	F	Y	P	G	P	G	H	T	P	D	N	V	V	V	W	L	P	E	N	R	V	L	F	G	G	C	F	V	K	P
IMP-16	K	N	K	I	E	I	F	Y	P	G	P	G	H	T	Q	D	N	V	V	V	W	L	P	E	K	K	I	L	F	G	G	C	F	V	K	P
IMP-18	K	N	K	I	E	V	F	Y	P	G	P	G	H	T	Q	D	N	V	V	V	W	L	P	E	K	K	I	L	F	G	G	C	F	V	K	P
IMP-19	K	N	K	I	E	V	F	Y	P	G	P	G	H	T	Q	D	N	V	V	V	W	L	P	E	K	K	I	L	F	G	G	C	F	V	K	P
IMP-20	K	N	K	I	E	V	F	Y	P	G	P	G	H	T	Q	D	N	V	V	V	W	L	P	E	K	K	I	L	F	G	G	C	F	V	K	P
IMP-21	K	N	K	I	E	V	F	Y	P	G	P	G	H	T	Q	D	N	V	V	V	W	L	P	K	N	K	I	L	F	G	G	C	F	V	K	P
IMP-22	K	N	K	I	E	V	F	Y	P	G	P	G	H	T	Q	D	N	V	V	V	W	L	P	E	K	K	I	L	F	G	G	C	F	V	K	P
IMP-24	K	N	K	I	E	V	F	Y	P	G	P	G	H	T	Q	D	N	V	V	V	W	L	P	E	K	K	I	L	F	G	G	C	F	V	K	P
IMP-25	K	N	K	I	E	V	F	Y	P	G	P	G	H	T	P	D	N	V	V	V	W	L	P	E	R	K	I	L	F	G	G	C	F	I	K	P
IMP-26	K	N	K	I	E	V	F	Y	P	G	P	G	H	T	P	D	N	L	V	V	W	L	P	E	R	K	I	L	F	G	G	C	F	I	K	P
IMP-27	K	D	K	I	E	V	F	Y	P	G	P	G	H	T	Q	D	N	V	V	V	W	L	P	E	K	E	I	L	F	G	G	C	F	V	K	P
IMP-28	K	N	K	I	E	V	F	Y	P	G	P	G	H	T	P	D	N	V	V	V	W	L	P	E	N	R	V	L	F	G	G	C	F	V	K	P
IMP-29	K	K	K	I	E	V	F	Y	P	G	P	G	H	T	P	D	N	V	V	V	W	L	P	E	N	R	V	L	F	G	G	C	F	V	K	P
IMP-30	K	N	K	I	E	V	F	Y	P	G	P	G	H	T	P	D	N	V	V	V	W	L	P	E	R	K	I	L	F	G	G	C	F	I	K	P
IMP-31	K	H	K	I	E	V	F	Y	P	G	P	G	H	T	Q	D	N	V	V	V	W	L	P	E	K	K	I	L	F	G	G	C	F	I	K	P
IMP-32	K	N	K	I	E	V	F	Y	P	G	P	G	H	T	Q	D	N	V	V	V	W	L	P	E	K	K	I	L	F	G	G	C	F	V	K	P
IMP-33	K	N	K	I	E	V	F	Y	P	G	P	G	H	T	Q	D	N	L	V	V	W	L	P	E	S	K	I	L	F	G	G	C	F	V	K	P
IMP-34	K	N	K	I	E	V	F	Y	P	G	P	G	H	T	P	D	N	V	V	V	W	L	P	E	R	K	I	L	F	G	G	C	F	I	K	P
IMP-35	K	H	K	I	E	V	F	Y	P	G	P	G	H	T	Q	D	N	V	V	V	W	L	P	E	K	K	I	L	F	G	G	C	F	I	K	P
IMP-37	K	N	K	I	E	V	F	Y	P	G	P	G	H	T	Q	D	N	L	V	V	W	L	P	E	S	K	I	L	F	G	G	C	F	I	K	P
IMP-38	K	N	K	I	E	V	F	Y	P	G	P	G	H	T	P	D	N	L	V	V	W	L	P	E	R	K	I	L	F	G	G	C	F	I	K	P
IMP-40	K	N	K	I	E	V	F	Y	P	G	P	G	H	T	P	D	N	V	V	V	W	L	P	E	R	K	I	L	F	G	G	C	F	I	K	P
IMP-41	K	N	K	I	E	V	F	Y	P	G	P	G	H	T	Q	D	N	V	V	V	W	L	P	K	N	K	I	L	F	G	G	C	F	V	K	P
IMP-42	K	N	K	I	E	V	F	Y	P	G	P	G	H	T	P	D	N	V	V	V	W	L	P	E	R	K	I	L	F	G	G	C	F	I	K	P
IMP-43	K	K	K	I	E	I	F	Y	P	G	P	G	H	T	P	D	N	V	V	V	W	L	P	E	H	R	V	L	F	G	G	C	F	V	K	P
IMP-44	K	N	K	I	E	V	F	Y	P	G	P	G	H	T	Q	D	N	V	V	V	W	L	P	K	N	K	I	L	F	G	G	C	F	V	K	P

Gray: Signal peptide; Red: Zn(II) ligands; Green: positions related to substrate preferences; Ligh Blue: positions that tolerate variations with minnor susbtrate dependance

IMP variants protein sequence alignment- BBL numbering

	227	228	231	232	233	234	235	236	237	238	241	242	243	244	245	246	247	248	249	250	251	252	253	254	a	b	255	256	257	258	259	260	261	262	263	264
IMP-1	Y	G	L	G	N	L	G	D	A	N	I	E	A	W	P	K	S	A	K	L	L	K	S	K	Y	G	K	A	K	L	V	V	P	S	H	S
IMP-2	D	G	L	G	N	L	G	D	A	N	L	E	A	W	P	K	S	A	K	I	L	M	S	K	Y	V	K	A	K	L	V	V	S	S	H	S
IMP-3	Y	G	L	G	N	L	G	D	A	N	I	E	A	W	P	K	S	A	K	L	L	K	S	K	Y	G	K	A	K	L	V	V	P	G	H	S
IMP-4	Y	G	L	G	N	L	G	D	A	N	L	E	A	W	P	K	S	A	K	L	L	I	S	K	Y	G	K	A	K	L	V	V	P	S	H	S
IMP-5	Y	G	L	G	N	L	G	D	A	N	V	E	A	W	P	K	S	A	K	L	L	M	S	K	Y	G	K	A	K	L	V	V	P	S	H	S
IMP-6	Y	G	L	G	N	L	G	D	A	N	I	E	A	W	P	K	S	A	K	L	L	K	S	K	Y	G	K	A	K	L	V	V	P	G	H	S
IMP-7	Y	G	L	G	N	L	G	D	A	N	L	E	A	W	P	K	S	A	K	L	L	V	S	K	Y	G	K	A	K	L	V	V	P	S	H	S
IMP-8	D	G	L	G	N	L	G	D	A	N	L	E	A	W	P	K	S	A	K	I	L	M	S	K	Y	G	K	A	K	L	V	V	S	S	H	S
IMP-9	Y	G	L	G	N	L	G	D	A	N	L	E	A	W	P	K	S	A	K	L	L	M	S	K	Y	S	K	A	K	L	V	V	P	S	H	S
IMP-10	Y	G	L	G	N	L	G	D	A	N	I	E	A	W	P	K	S	A	K	L	L	K	S	K	Y	G	K	A	K	L	V	V	P	S	H	S
IMP-11	Y	G	L	G	N	L	D	D	A	N	V	E	A	W	P	H	S	A	E	K	L	I	S	K	Y	G	N	A	K	L	V	V	P	S	H	S
IMP-12	D	G	L	G	N	L	D	D	A	N	L	K	A	W	P	K	S	A	K	I	L	M	S	K	Y	G	K	A	K	L	V	V	S	G	H	S
IMP-13	H	G	L	G	N	L	G	D	A	N	L	E	A	W	P	K	S	A	K	I	L	M	S	K	Y	G	K	A	K	L	V	V	S	S	H	S
IMP-14	D	G	L	G	Y	L	G	D	A	N	L	E	A	W	P	K	S	A	K	I	L	M	S	K	Y	G	K	A	K	L	V	V	S	S	H	S
IMP-15	Y	G	L	G	N	L	G	D	A	N	L	E	A	W	P	K	S	A	K	I	L	M	S	K	Y	G	K	A	K	L	V	V	S	S	H	S
IMP-16	Y	G	L	G	N	L	D	D	A	N	V	E	A	W	P	H	S	A	E	I	L	M	S	R	Y	G	N	A	K	L	V	V	P	S	H	S
IMP-18	D	G	L	G	N	L	G	D	A	N	L	E	A	W	P	K	S	A	K	I	L	M	S	K	Y	G	K	A	K	L	V	V	S	S	H	S
IMP-19	D	G	L	G	N	L	G	D	A	N	L	E	A	W	P	K	S	A	K	I	L	M	S	K	Y	V	K	A	K	L	V	V	S	S	H	S
IMP-20	D	G	L	G	N	L	G	D	A	N	L	E	A	W	P	K	S	A	K	I	L	M	S	K	Y	V	K	A	K	L	V	V	S	S	H	S
IMP-21	Y	G	L	G	N	L	D	D	A	N	V	E	A	W	P	H	S	A	E	K	L	I	S	K	Y	G	N	A	K	L	V	V	P	S	H	S
IMP-22	Y	G	L	G	N	L	D	D	A	N	V	V	A	W	P	H	S	A	E	I	L	M	S	R	Y	G	N	A	K	L	V	V	P	S	H	S
IMP-24	D	G	L	G	N	L	G	D	A	N	L	E	A	W	P	K	S	A	K	I	L	M	S	K	Y	G	K	A	K	L	V	V	S	S	H	S
IMP-25	Y	G	L	G	N	L	S	D	A	N	I	E	A	W	P	K	S	A	K	L	L	K	S	K	Y	G	K	A	K	L	V	V	P	G	H	S
IMP-26	Y	G	L	G	N	L	G	D	A	N	L	E	A	W	P	K	S	A	K	L	L	I	S	K	Y	G	K	A	K	L	V	V	P	S	H	S
IMP-27	H	G	L	G	N	L	G	D	A	N	L	E	A	W	P	E	S	A	K	I	L	M	E	K	Y	G	K	A	K	L	V	V	S	G	H	S
IMP-28	Y	G	L	G	N	L	G	D	A	N	L	E	A	W	P	K	S	A	K	L	L	M	S	K	Y	G	K	A	K	L	V	V	P	S	H	S
IMP-29	Y	G	L	G	N	L	D	D	A	N	V	E	A	W	P	H	S	A	E	I	L	M	S	R	Y	G	N	A	K	L	V	V	P	S	H	S
IMP-30	Y	G	L	G	N	L	G	D	A	N	I	E	A	W	P	K	S	A	K	L	L	K	S	K	Y	G	K	A	K	L	V	V	P	S	H	S
IMP-31	D	G	L	G	Y	L	G	D	A	N	L	E	A	W	P	K	S	A	E	T	L	M	S	K	Y	G	N	A	K	L	V	V	S	S	H	S
IMP-32	D	G	L	G	Y	L	G	D	A	N	L	E	A	W	P	K	S	A	K	I	L	M	S	K	Y	G	K	A	K	L	V	V	S	S	H	S
IMP-33	H	G	L	G	N	L	G	D	A	N	L	E	A	W	P	K	S	A	K	I	L	M	S	K	Y	G	K	A	K	L	V	V	S	S	H	S
IMP-34	Y	G	L	G	N	L	G	D	A	N	I	E	A	W	P	K	S	A	K	L	L	K	S	K	Y	G	K	A	K	L	V	V	P	S	H	S
IMP-35	D	G	L	G	Y	L	G	D	A	N	L	E	A	W	P	K	S	A	E	T	L	M	S	K	Y	G	N	A	K	L	V	V	S	S	H	S
IMP-37	H	G	L	G	N	L	G	D	A	N	L	E	A	W	P	K	S	A	K	I	L	M	S	K	Y	G	K	A	K	L	V	V	S	S	H	S
IMP-38	Y	G	L	G	N	L	G	D	A	N	L	E	A	W	P	K	S	A	K	L	L	I	S	K	Y	G	K	A	K	L	V	V	P	G	H	S
IMP-40	Y	G	L	G	N	L	G	D	A	N	I	E	A	W	P	K	S	A	K	L	L	K	S	K	Y	G	K	A	K	L	V	V	P	S	H	S
IMP-41	Y	G	L	G	N	L	D	D	A	N	V	E	A	W	P	H	S	A	E	K	L	I	S	K	Y	G	N	A	K	L	V	V	P	S	H	S
IMP-42	Y	G	L	G	N	L	G	D	A	N	I	E	A	W	P	K	S	A	K	L	L	K	S	K	Y	G	K	A	K	L	V	V	P	S	H	S
IMP-43	Y	G	L	G	N	L	G	D	A	N	L	E	A	W	P	K	S	A	K	L	L	V	S	K	Y	G	K	A	K	L	V	V	P	S	H	S
IMP-44	Y	G	L	G	N	L	D	D	A	N	V	E	A	W	P	H	S	A	E	K	L	I	S	K	Y	G	N	A	K	L	V	V	P	S	H	S

Gray: Signal peptide; Red: Zn(II) ligands; Green: positions related to substrate preferences; Ligh Blue: positions that tolerate variations with minnor susbtrate dependance

IMP variants protein sequence alignment- BBL numbering

	265	266	295	296	297	298	299	300	301	302	303	304	305	306	307	308	309	310	311	312	313	314	315	316	317	318	319	320	321	322		
IMP-1	E	V	G	D	A	S	L	L	K	L	T	L	E	Q	A	V	K	G	L	N	E	S	K	K	P	S	K	P	S	N	*	
IMP-2	E	I	G	D	A	S	L	L	K	R	T	W	E	Q	A	V	K	G	L	N	E	S	K	K	P	S	Q	P	S	N	*	
IMP-3	E	V	G	D	A	S	L	L	K	L	T	L	E	Q	A	V	K	G	L	N	E	S	K	K	P	S	K	P	S	N	*	
IMP-4	E	A	G	D	A	S	L	L	K	L	T	L	E	Q	A	V	K	G	L	N	E	S	K	K	P	S	K	L	S	N	*	
IMP-5	E	V	G	D	A	S	L	L	K	R	T	L	E	Q	A	V	K	G	L	N	E	S	K	K	P	S	K	P	S	N	*	
IMP-6	E	V	G	D	A	S	L	L	K	L	T	L	E	Q	A	V	K	G	L	N	E	S	K	K	P	S	K	P	S	N	*	
IMP-7	E	V	G	D	A	S	L	L	K	R	T	L	E	Q	A	V	K	G	L	N	E	S	K	K	L	S	K	P	S	N	*	
IMP-8	E	I	G	D	A	S	L	L	K	R	T	W	E	Q	A	V	K	G	L	N	E	S	K	K	P	S	Q	P	S	N	*	
IMP-9	D	I	G	D	S	S	L	L	K	L	T	W	E	Q	T	V	K	G	F	N	E	S	K	K	S	T	T	A	H	*	-	
IMP-10	E	V	G	D	A	S	L	L	K	L	T	L	E	Q	A	V	K	G	L	N	E	S	K	K	P	S	K	P	S	N	*	
IMP-11	D	I	G	D	A	S	L	L	K	L	T	W	E	Q	A	V	K	G	L	N	E	S	K	K	S	N	T	V	H	*	-	
IMP-12	E	I	G	N	A	S	L	L	K	L	T	W	E	Q	A	V	K	G	L	K	E	S	K	K	P	L	L	P	S	N	*	
IMP-13	E	K	G	D	A	S	L	M	K	R	T	W	E	Q	A	L	K	G	L	K	E	S	K	K	T	S	S	P	S	N	*	
IMP-14	D	I	G	D	V	S	L	L	K	R	T	W	E	Q	A	V	K	G	L	N	E	S	K	K	S	S	Q	P	S	D	*	
IMP-15	E	T	G	N	A	S	L	L	K	L	T	W	E	Q	A	V	K	G	L	K	E	S	K	K	P	S	L	P	S	N	*	
IMP-16	D	V	G	D	A	S	L	L	K	L	T	W	E	Q	A	V	K	G	L	K	E	S	K	K	P	S	Q	P	S	N	*	
IMP-18	E	I	G	N	A	S	L	L	Q	R	T	W	E	Q	A	V	K	G	L	N	E	S	K	K	P	L	Q	P	S	S	*	
IMP-19	E	I	G	D	A	S	L	L	K	R	T	W	E	Q	A	V	K	G	L	N	E	S	K	K	P	S	Q	P	S	N	*	
IMP-20	E	I	G	D	A	S	L	L	K	R	T	W	E	Q	A	V	K	G	L	N	E	S	K	K	P	S	Q	P	S	N	*	
IMP-21	D	I	G	D	A	S	L	L	K	L	T	W	E	Q	A	V	K	G	L	N	E	S	K	K	S	N	T	V	H	*	-	
IMP-22	D	I	G	D	A	S	L	L	K	L	T	W	E	Q	A	V	K	G	L	K	E	S	K	K	P	S	E	P	S	N	*	
IMP-24	E	I	G	D	A	S	L	L	K	R	T	W	E	Q	A	V	K	G	L	N	E	S	R	K	P	S	Q	P	S	N	*	
IMP-25	E	V	G	D	A	S	L	L	K	L	T	L	E	Q	A	V	K	G	L	N	E	S	K	K	P	S	K	P	S	N	*	
IMP-26	E	A	G	D	A	S	L	L	K	L	T	L	E	Q	A	V	K	G	L	N	E	S	K	K	P	S	K	L	S	N	*	
IMP-27	E	T	G	D	A	T	H	L	K	R	T	W	E	Q	A	V	K	G	L	K	E	S	K	K	T	L	Q	P	S	N	*	
IMP-28	E	V	G	D	A	S	L	L	K	R	T	L	E	H	A	V	K	G	L	N	E	S	K	K	P	S	K	P	S	N	*	
IMP-29	D	I	G	N	A	S	L	L	K	L	T	W	E	Q	A	V	K	G	L	K	E	S	K	K	P	S	Q	P	S	N	*	
IMP-30	E	V	G	D	A	S	L	L	K	L	T	L	E	Q	A	V	K	G	L	N	E	S	K	K	P	S	K	P	S	N	*	
IMP-31	E	I	G	G	A	S	L	L	K	R	T	W	E	Q	A	V	K	G	L	K	E	S	K	N	H	H	S	P	K	*	-	
IMP-32	D	I	G	D	V	S	L	L	K	R	T	W	E	Q	A	V	K	G	L	N	E	S	K	K	S	S	Q	P	S	D	*	
IMP-33	E	K	G	D	A	S	L	L	K	R	T	W	E	Q	A	L	K	G	L	K	E	S	K	K	T	S	S	P	S	N	*	
IMP-34	E	V	G	D	A	S	L	L	K	L	T	L	E	Q	A	V	K	G	L	N	E	S	K	K	P	S	K	P	S	N	*	
IMP-35	E	I	G	G	A	S	L	L	K	R	T	W	E	Q	A	V	K	G	L	K	E	S	K	K	P	S	Q	P	N	N	*	
IMP-37	E	K	G	D	A	S	L	M	K	R	T	W	E	Q	A	L	K	G	L	K	E	S	K	K	T	S	S	Q	S	T	A	S
IMP-38	E	A	G	D	A	S	L	L	K	L	T	L	E	Q	A	V	K	G	L	N	E	S	K	K	P	S	K	L	S	N	*	
IMP-40	E	V	G	D	A	S	L	L	K	L	T	L	E	Q	A	V	K	G	L	N	E	S	K	K	P	S	K	P	S	N	*	
IMP-41	D	I	G	D	A	S	L	L	K	L	T	W	E	Q	A	V	K	G	L	N	E	S	K	K	S	N	T	V	H	*	-	
IMP-42	E	V	G	D	A	S	L	L	K	L	T	L	E	Q	A	V	K	G	L	N	E	S	K	K	P	S	K	P	S	N	*	
IMP-43	E	V	G	D	A	S	L	L	K	R	T	L	E	Q	A	V	K	G	L	N	E	S	K	K	L	S	K	P	S	N	*	
IMP-44	D	I	G	D	A	S	L	L	K	L	T	W	E	Q	A	V	K	G	L	N	E	S	K	K	S	N	T	V	H	*	-	

Gray: Signal peptide; Red: Zn(II) ligands; Green: positions related to substrate preferences; Ligh Blue: positions that tolerate variations with minnor susbtrate dependance
